# *Akkermansia muciniphila* outer membrane protein Amuc_0904 modulates intestinal homeostasis by promoting goblet cell differentiation

**DOI:** 10.1080/19490976.2025.2587405

**Published:** 2025-12-22

**Authors:** Liu Yang, Jianming Yang, Xueting Kong, Qiang Tang, Ruofan Song, Kaichen Zhou, Xiao-Jing Quan, Qiang Zhang, Yajie Zhang, Chunze Zhang, Geng Pei, Chunhui Miao, Kaiyuan Yu, Zhi-Song Zhang, Zhi Yao, Quan Wang

**Affiliations:** a Tianjin Institute of Immunology, Key Laboratory of Immune Microenvironment and Disease (Ministry of Education), School of Basic Medical Sciences, Tianjin Medical University, State Key Laboratory of Experimental Hematology, Chinese Academy of Medical Sciences and Peking Union Medical College, Tianjin, People's Republic of China; b State Key Laboratory of Medicinal Chemical Biology and College of Pharmacy, Tianjin Key Laboratory of Molecular Drug Research, Nankai University, Tianjin, People's Republic of China; c Department of General Surgery, Tianjin Beichen Hospital, Tianjin, People's Republic of China; d Department of Colorectal Surgery, Tianjin Union Medical Center, Tianjin Institute of Coloproctology, Tianjin, China; e Department of Pathology, Tianjin Medical University Cancer Institute and Hospital, National Clinical Research Center of Cancer, Key Laboratory of Cancer Prevention and Therapy, Tianjin, Tianjin's Clinical Research Center of Cancer, Tianjin, People's Republic of China; f Institute of Medicinal Biotechnology, Chinese Academy of Medical Sciences and Peking Union Medical College, State Key Laboratory of Experimental Hematology, Beijing, People's Republic of China

**Keywords:** *Akkermansia muciniphila*, Amuc_0904, goblet cell, MET, intestinal homeostasis

## Abstract

*Akkermansia muciniphila* is recognized as a promising probiotic that improves the symptoms of a variety of diseases. However, the role and mechanism of *A. muciniphila* in regulating intestinal homeostasis remain to be explored. Here, we discovered that *A. muciniphila* was dramatically increased during colitis recovery, and its colonization greatly increased goblet cells to protect the intestinal barrier in mice. Amuc_0904, a previously uncharacterized *A. muciniphila* outer membrane protein, was identified to induce goblet cell differentiation. Mechanistically, Amuc_0904 directly interacted with MET and decreased its phosphorylation in epithelia, leading to decreased Wnt/β-catenin signaling and enhanced oxidative phosphorylation and mitochondrial function. Furthermore, Amuc_0904 and engineered probiotic *Escherichia coli* Nissle 1917 expressing Amuc_0904 were demonstrated to protect mice from colitis and colitis-associated colorectal cancer. The study reveals a previously unknown mechanism of *A. muciniphila*-mediated intestinal homeostasis recovery and provides a bioactive molecule with the potential to treat intestinal disorders.

## Introduction

The gut microbiota is a complex ecological community that affects both normal physiology and disease susceptibility.^1^^,^^2^ The perturbations in the composition and activity of the gut microbiota during colitis and recovery have been constantly observed,^3^^,^^4^ and certain microorganisms are associated with intestinal inflammation remission.^5-9^ However, the mechanism through which commensal gut microbiota drive colitis remission remains to be investigated.

*Akkermansia muciniphila*, an anaerobic Gram-negative bacterium, has emerged as a potential probiotic agent.^10^^,^^11^ Accumulating evidence suggests that *A. muciniphila* improves obesity, diabetes, hepatic steatosis, inflammation, and various cancers in both mouse and human models,^12-15^ with some of its metabolites and bioactive components considered to be responsible for these benefits.^16-20^ *A. muciniphila* is well known to be an exclusive mucin-degrading bacterium; however, a few studies revealed that administration of *A. muciniphila* led to the generation of mucin-secreting goblet cells and increased mucus thickness.^2^ The specific components and mechanisms by which *A. muciniphila* induces goblet cell and mucin production are currently unknown.

MET, also known as hepatocyte growth factor receptor (HGFR), is a tyrosine kinase receptor consisting of a 50 kDa extracellular *α*-chain and a 145 kDa transmembrane *β*-chain, which is primarily expressed in epithelial cells.^21^ Upon binding to its ligand HGF, two MET subunits dimerize and undergo autophosphorylation. This process leads to the subsequent activation of the Ras/Raf, PI3K/Akt/mTOR, and STAT3 pathways.^22^ The MET receptor plays a significant role in various physiological and pathological processes such as embryonic development, tissue repair, tumorigenesis, and metastasis.^23^^,^^24^ The expression of MET has been demonstrated to be ubiquitous in the colorectum, and it plays a role in regulating intestinal homeostasis and regeneration, as well as adenoma formation.^25^ MET expression is elevated in colonic biopsies from patients with active ulcerative colitis, as well as in those with IBD-associated dysplasia and cancer.^26^^,^^27^ Nevertheless, its impact on goblet cells is unknown.

In this study, we revealed that *A. muciniphila* was significantly increased during the phases of inflammatory remission. A novel outer membrane (OM) protein, Amuc_0904, of *A. muciniphila*, has been discovered to promote goblet cell differentiation by interacting with the MET protein. Collectively, these findings reveal a previously unknown mechanism that accounts for the modulation of *A. muciniphila* on intestinal homeostasis and provides a bioactive molecule derived from commensal bacteria for reshaping intestinal homeostasis.

## 2 Materials and methods

### 2.1 Mice

Male C57BL/6J mice (6–8 weeks old) were obtained from the Academy of Military Medical Science (Beijing, China). Animals were maintained under specific pathogen-free (SPF) conditions in a barrier facility with a 12 h light/dark cycle at the Department of Laboratory Animal Science and Technology, Tianjin Medical University. All animal procedures were approved by the Animal Care and Use Committee of Tianjin Medical University (TMUaMEC 2023017).

### 2.2 Human samples

Colonic tissues from ulcerative colitis patients were obtained from Tianjin Union Medical Center. Colorectal cancer (CRC) samples were collected from resection surgeries performed at Tianjin Medical University Cancer Institute and Hospital. All participants provided informed consent, and relevant clinical information is summarized in Table S1. The study protocols were approved by the Ethics Committee of Tianjin Union Medical Center (2023-B30) and the Ethics Committee of Tianjin Medical University Cancer Institute and Hospital (bc2023182).

### 2.3 Cell lines

The human colorectal cancer cell lines HT−29 (ATCC HTB−38), LS174T (ATCC CL−188), and Caco2 (ATCC HTB−37) were acquired from the American Type Culture Collection (ATCC). Cells were cultured in Dulbecco’s Modified Eagle Medium (DMEM) supplemented with 10% fetal bovine serum (FBS), and maintained at 37 °C in a humidified atmosphere containing 5% CO₂.

### 2.4 Bacterial strains

*Akkermansia muciniphila*, *Bacteroides uniformis*, *B. intestinalis*, *B. oleiciplenus*, and *Prevotella clara* were isolated from fecal samples of DSS-treated mice after fecal microbiota transplantation (day 22). Strains were cultured anaerobically using an anaerobic bottle system at 37 °C for 24 h in modified Gifu Anaerobic Medium (GAM) supplemented with 0.1% L-cysteine, 2% vitamin K1, 0.1% resazurin, and 0.5% hemoglobin.

*Escherichia coli* K12 and *E. coli* Nissle 1917 (EcN) were purchased from Beijing Dingguo Biotechnology Development Center and Beijing Biobw Biotechnology Company, respectively. These strains were cultured aerobically in Luria-Bertani (LB) broth at 37 °C for 12 h.

### 2.5 Cell treatment

Cells were seeded in 24-well plates and allowed to adhere overnight prior to stimulation. For bacterial stimulation, *A. muciniphila* and *B. uniformis* were grown anaerobically, harvested by centrifugation (4,500 × g, 10 min), and resuspended in PBS containing 0.1% L-cysteine. Cells were infected at a multiplicity of infection (MOI) of 2 for cell line-specific durations: Caco2 (15 h), LS174T (48 h), HT−29 (72 h). Sterile PBS with 0.1% L-cysteine served as the control.

For supernatant treatment, filter-sterilized (0.22 μm) culture supernatants were applied at 50% v/v (Caco2) or 20% v/v (HT−29, LS174T). The bacterial pellets were inactivated via autoclaving (121 °C, 20 min) or pasteurization (70 °C, 30 min).

For protein stimulation assays, outer membrane proteins (OMPs) were extracted using a Bacterial Outer Membrane Protein Extraction Kit (BestBio, China, BB−31512). Cells were treated with OMPs at 0.5 μg/mL (Caco−2, 10 h) or 5 μg/mL (HT−29, 72 h; LS174T, 48 h). Purified recombinant proteins were applied as specified.

For inhibitors/agonists treatment, CHIR−99021 (Wnt agonist, 10 μM), DAPT (Notch inhibitor, 5 μM), and capmatinib (MET inhibitor, 10 nM) were dissolved in DMSO and applied for the indicated durations.

### 2.6 Immunofluorescent staining

For tissue sections, fresh colon samples were embedded in OCT, sectioned at 5 μm, and fixed in pre-cooled acetone. After peroxidase quenching with 3% H₂O₂ in methanol and blocking with 5% BSA, sections were incubated with primary antibodies (MUC2, *β*-catenin, *p*-MET, CD45, Epcam, MET, FLAG) at 4 °C overnight, followed by Alexa Fluor-conjugated secondary antibodies. Nuclei were stained with DAPI. Images were acquired using a Leica TCS-SP8 confocal microscope (Leica Microsystems, Germany).

For cells and organoids, cells (HT−29, LS174T, Caco2) and colonic organoids grown on coverslips were fixed in 4% PFA, permeabilized with 0.25% Triton X−100, blocked with 5% BSA, and incubated with primary antibodies at 4 °C overnight. Secondary antibodies (AF488/AF594) were applied for 2 h. After DAPI staining, samples were mounted and imaged.

For bacterial staining, EcN cultures were induced with 0.5% arabinose, fixed in 2% PFA, and air-dried on slides. After permeabilization (0.1% Triton X−100) and blocking (5% goat serum), samples were incubated with anti-FLAG antibody, followed by AF594-conjugated secondary antibody. Membranes were labeled with WGA (0.1 mg/mL), and nuclei stained with DAPI before imaging.

### 2.7 Hematoxylin and eosin (H&E) and AB-PAS staining

Paraffin-embedded tissues were sectioned at 3 μm, deparaffinized, and stained with H&E or AB-PAS using commercial kits (Solarbio, China, G1285). H&E images were acquired using an Olympus BX46 microscope. Goblet cells were quantified by counting cells in five random high-power fields (HPF).

### 2.8 Immunohistochemistry (IHC)

Deparaffinized sections underwent antigen retrieval in citrate (pH 6.0) or EDTA (pH 9.0) buffer. Endogenous peroxidase was quenched with 3% H₂O₂. After blocking with 5% goat serum, sections were incubated with primary antibodies (MUC2, Ki67, Occludin, Cleaved caspase−3, *p*-MET) at 4 °C overnight, followed by HRP-labeled secondary antibody and DAB development. Sections were counterstained with hematoxylin, dehydrated, and imaged using an Olympus BX46 microscope. Staining intensity was quantified with Image Pro Plus v6.0.

### 2.9 Intestinal Organoid Culture and Treatment

Colonic crypts were isolated from proximal colon segments using Gentle Cell Dissociation Reagent (GCDR, StemCell, Canada, 100−0485), filtered through a 70-μm strainer, and embedded in Matrigel (Corning, America, 356234). Organoids were cultured in IntestiCult™ Organoid Growth Medium (StemCell, Canada, 06005) at 37 °C with 5% CO₂, with medium changes every 48–72 h.

For treatments, organoids were exposed to capmatinib (20 nM), CHIR−99021 (10 μM), oligomycin (5 μM), and/or Amuc_0904 (0.5 μM) for 72 h or 96 h. For ROS assays, organoids were primed with LPS (10 μg/mL, 48 h) before co-treatment. Organoid images were captured using an Olympus TH4−200 microscope; budding and formation efficiency were quantified 6−8 d after seeding 150 crypts.

For passaging, organoids were dissociated with GCDR, then re-embedded as above.

### 2.10 Antibiotic treatment

To deplete gut microbiota, mice received oral administration of a cocktail of antibiotics (AVMN, 1 g/L ampicillin, 1 g/L neomycin, 1 g/L metronidazole, 500 mg/L vancomycin) in drinking water *ad libitum* for 5−7 d. Antibiotic solutions were refreshed every 72−96 h to ensure potency.

### 2.11 Fecal microbiota transplantation (FMT)

Fecal samples from six healthy volunteers were homogenized in sterile PBS containing 30% glycerol and 2.5% L-cysteine, centrifuged (800 × g, 3 min), and filtered through 70-μm membranes. Recipient mice received an AVMN antibiotic cocktail for 7 d, followed by daily intragastric administration of 200 μL microbiota suspension for 9 d.

### 2.12 DSS-induced colitis mouse model

For acute colitis recovery model, mice were pretreated with AVMN antibiotics for 7 d and human FMT for 9 d, then administered 1% DSS for 22 d. Microbiota and tissues were collected on days 0, 8, and 22 for 16S sequencing and histology.

For bacterial intervention mode, following a 5-d pretreatment with the AVMN antibiotic cocktail, mice received daily oral gavage of individual bacterial strains (5 × 10⁸ CFU) for 5 d. Subsequently, colitis and systemic inflammation were induced by administering 2.5% DSS in drinking water alongside intraperitoneal injection of LPS (8 mg/kg, i.p.) for 6 d, during which bacterial administration was continued. Disease activity index (DAI) was scored based on weight loss, bleeding, and stool consistency.

For protein treatment model, mice received daily oral gavage of 10 μg Amuc_0904 in 2.5% glycerol-PBS (vehicle control: PBS with 2.5% glycerol alone).

Colon tissues were fixed in 4% PFA for histology or embedded in OCT for frozen sections.

### 2.13 Bacterial isolation and identification

Fecal samples were homogenized in PBS with 0.1% L-cysteine, serially diluted, and plated on MRS, BHI, and blood agar plates. Colonies were cultured anaerobically, and genomic DNA was extracted using a TIANamp Bacteria DNA Kit (TIANGEN, China, DP302) for full-length 16S rRNA PCR amplification and sequencing (primers 27F/1492R). Taxonomy was assigned using the Ezbiocloud database.

### 2.14 16S rRNA gene sequencing

Fecal DNA was extracted using a Stool DNA Kit (Omega, America, D4015). 16S sequencing was performed by Majorbio Bio-Pharm Technology Co., Ltd. (Shanghai, China) according to standard manufacturer instructions. Data were analyzed on the Majorbio I-Sanger Cloud Platform (alpha/beta diversity, taxonomy). Raw data are available under NCBI SRA accessions PRJNA116076 and PRJNA1105550.

### 2.15 Bacterial whole genome sequencing

Genomic DNA from *A. muciniphila* TMU was extracted using Bacteria DNA Kit (Tiangen, China, DP302) according to the manufacturer’s protocol and quantified using a TBS−380 fluorometer (Turner BioSystems Inc., Sunnyvale, CA). Whole-genome sequencing employed a hybrid strategy combining PacBio RS long-read technology and Illumina NovaSeq 6000 sequencing (Shanghai BIOZERON Co., Ltd.). Assembly used ABySS (http://www.bcgsc.ca/platform/bioinfo/software/abyss) and Canu (https://github.com/marbl/canu), with GapCloser (https://sourceforge.net/projects/soapdenovo2/files/GapCloser/) for polishing. The complete genome is deposited under GenBank accession CP156688.

### 2.16 RNA extraction and qRT-PCR

Total RNA was extracted from Caco2, HT−29, and LS174T cells, organoids, and colon tissues using a Total RNA Extraction Kit (Solarbio, China, R1200). cDNA was synthesized from the extracted RNA using the RevertAid First Strand cDNA Synthesis Kit (Thermo Fisher Scientific, America, K1621). qPCR was performed using Ultra SYBR Mixture on an Applied Biosystems QuantStudio 1 Real-Time PCR System under the following conditions: initial denaturation at 95 °C for 10 min; 35 cycles of 95 °C for 15 s, 60 °C for 30 s, and 72 °C for 30 s. Relative gene expression was calculated using the 2^−ΔΔCt^ method, with GAPDH or *β*-actin as the internal reference.

### 2.17 RNA sequencing

Total RNA was isolated from HT−29 cells using TRIzol Reagent. RNA sequencing was conducted by Majorbio Bio-Pharm Technology Co. Ltd. according to standard procedures. Raw paired-end reads were processed as previously described.^28^ The complete dataset is accessible in the NCBI GEO database under accession number GSE288821.

### 2.18 RNA interference

siRNAs targeting MET (si*MET*) and a scrambled control (siScr) were purchased from GenePharma (Shanghai, China). HT−29 cells were transfected using Lipofectamine 3000 for 48 h. Knockdown efficiency was assessed by qRT-PCR and western blotting.

### 2.19 Recombinant protein purification

Recombinant His-FLAG-tagged proteins (Amuc_0032, Amuc_0074, Amuc_0610, Amuc_0682, Amuc_0687, Amuc_0820, Amuc_0904, Amuc_1026) were expressed in *E. coli* BL21 (DE3) using the pET-28a ( + ) vector. Protein expression was induced with 100 μM IPTG at 16 °C for 18 h. Cells were lysed, and soluble proteins were purified by Ni-IDA affinity chromatography. Proteins were dialyzed and concentrated using Amicon Ultra−15 centrifugal filters.

GST-tagged MET^927^ was expressed in Rosetta (DE3) using the pGEX4T−3 vector and induced with 100 μM IPTG at 25 °C for 8 h. The protein was purified using glutathione Sepharose 4B, dialyzed, and concentrated. Protein concentration was determined spectrophotometrically (NanoDrop One, Thermo Scientific) using a BCA assay kit (Thermo Fisher Scientific).

### 2.20 Molecular docking

The 3D structures of Amuc_0904, *E. coli* K12 AcnA, and MET were prepared by assigning bond orders, adding hydrogens, and removing water/cofactors. Energy minimization was performed with the OPLS_4 force field. Protein-protein docking was carried out using Schrödinger's Piper module. Structural visualization was performed using PyMOL software.

### 2.21 Plasmid construction and engineered bacterial preparation

An arabinose-inducible expression system was constructed using the pTOPO-araBAD plasmid. To target Amuc_0904 to the outer membrane, a fusion gene encoding FLAG-tagged Amuc_0904 and extracellular loop 2 of OmpX was cloned into this plasmid, yielding pTOPO-araBAD-Ompx−904 (BAD−904). Both the empty vector (BAD-vector) and BAD−904 plasmid were transformed into *Escherichia coli* Nissle 1917 (EcN). Protein expression was induced with 0.5% arabinose at 37 °C for 8 h and confirmed by immunofluorescence.

### 2.22 Mitochondrial function assays

Cellular oxygen consumption rate (OCR) was quantified using a Seahorse XF96 Extracellular Flux Analyzer (Seahorse Biosciences, MA, U.S.A.). Cells were pretreated with LPS (1 μg/mL, 36 h) and Amuc_0904 (0.3 μM, 12 h) in XF microplates. The mitochondrial stress test was performed by sequential injection of oligomycin (2 μM), FCCP (0.75 μM), and rotenone/antimycin A (5 μM).

Intracellular ROS was detected using a commercial Reactive Oxygen Species Assay Kit (Beyotime, China, S0033) according to manufacturer protocols with the DCFH-DA probe (1:1000 dilution) and analyzed by either flow cytometry (BD Biosciences, San Jose, CA, U.S.A.) or fluorescence microscopy (Leica TCS-SP8).

The mitochondrial membrane potential (MMP) was assessed using JC−1 dye (Beyotime, China, C2006). The fluorescence (red/green ratio) was measured to evaluate depolarization.

### 2.23 Western blotting

Cells were lysed in RIPA buffer containing protease and phosphatase inhibitors. Proteins were denatured, separated by SDS-PAGE, and transferred to nitrocellulose membranes. Blots were probed with antibodies against *β*-catenin, active *β*-catenin, MET, *p*-MET, and *β*-actin, followed by HRP-conjugated secondary antibodies. Signals were detected with chemiluminescent substrate and quantified using ImageJ software.

### 2.24 Far western blotting and protein identification

Cell surface proteins from Caco2 cells were extracted using the Membrane and Cytosol Protein Extraction Kit (Beyotime, China, P0033) according to manufacturer's protocol, separated by SDS-PAGE, and transferred to PVDF membranes. Membranes were incubated with FLAG-Amuc_0904, and interactions were detected using anti-FLAG antibody. Specific protein bands were excised for mass spectrometry analysis.

### 2.25 Immunoprecipitation (IP) assays

Purified FLAG-Amuc_0904 (5 μg) and MYC-MET^927^ (5 μg) were incubated together as previously described^29^ and with antibody-conjugated beads (anti-FLAG or anti-MYC) overnight at 4 °C. Complexes were washed, eluted, and analyzed by western blotting using anti-FLAG and anti-MYC antibodies.

### 2.26 Ni-IDA pull-down assay

Recombinant Amuc_0904 (100 μg) was conjugated to Ni-IDA resin for 4 h at 4 °C. After washing, the resin was incubated with MYC-tagged MET^927^ (300 μg) overnight at 4 °C. Following five washes, bound complexes were eluted with imidazole-containing buffer, denatured, and analyzed by SDS-PAGE and immunoblotting using anti-MYC or anti-FLAG antibodies.

### 2.27 In situ proximity ligation assay (PLA)

Caco2 cells were fixed, blocked, and incubated with anti-MET and anti-FLAG antibodies. PLA probes were applied, followed by ligation and amplification steps according to the Duolink® protocol (Sigma-Aldrich, America, DUO92014). Samples were mounted with DAPI-containing medium and imaged by confocal microscopy.

### 2.28 Mesenteric lymph nodes (MLNs) and colonic lamina propria cells isolation

MLNs were aseptically harvested, rinsed in ice-cold PBS, and mechanically dissociated through a 70-μm cell strainer using RPMI 1640 medium supplemented with 10% FBS. Isolated lymphocytes were pelleted by centrifugation, resuspended in PBS, and prepared for further fluorescence staining and flow cytometry analysis.

Colon tissues were cleaned, digested enzymatically (collagenase/DNase I/hyaluronic acid), filtered, and separated via Percoll gradient centrifugation as previously described.^28^ Pelleted leukocytes were washed in cold PBS and resuspended for evaluation.

### 2.29 Colonic intraepithelial lymphocytes (IELs) isolation

Freshly excised mouse colon tissues were immediately placed in ice-cold HBSS. After removal of adipose tissue and mesenteric lymph nodes, colons were opened longitudinally and luminal contents were flushed thoroughly with cold HBSS. The cleaned tissues were cut into 1−2 cm segments and incubated in 5 mL HBSS containing 5 mM EDTA and 2 mM DTT at 37 °C with shaking at 160 rpm for 30 min to dissociate epithelial cells. To improve IEL release, samples were vortexed briefly every 10 min during incubation. The supernatant containing released cells was filtered through a 70 μm nylon mesh and centrifuged to pellet the cells. For lymphocyte enrichment, cells were resuspended in 40% Percoll and centrifuged at 700 × g for 20 min at 4 °C (ramp rate: 5; brake: 0). Purified IELs were collected and washed twice with HBSS for subsequent applications.

### 2.30 Flow cytometry

Single-cell suspensions were stained for 30 minutes at 4 °C under light-protected conditions with fluorochrome-conjugated antibodies against: APC-anti-CD45, PE-Cy7-anti-CD11b, FITC-anti-F4/80, APC-Cy7-anti-CD45, PE-anti-MHCII, APC-anti-CD11c, PerCP/Cy5.5-anti-CD45, PE-anti-CD45, FITC-anti-CD3 and APC-anti-Ly6G.

For intracellular cytokine staining, cells were stimulated with Cell Activation Cocktail (with Brefeldin A, BioLegend, America, 423303) for 5 h at 37 °C. Prior to surface staining, viability was assessed using Zombie NIR™ Fixable Viability Kit (BioLegend, America, 423105). Surface immunostaining included FITC-anti-CD3 and PE-Cy7-anti-CD4. Following fixation and permeabilization according to manufacturer protocol, intracellular targets were stained for 30 min at 4 °C with: APC-anti-IL-17A, PerCP/Cy5.5-anti-IFN-*γ*, and PE-anti-IL−4.

For Treg and ILC3 cells analysis, Treg identification involved surface staining with FITC-anti-CD3, PE-Cy7-anti-CD4, and PE-anti-CD25, ILC3 identification involved surface staining with PerCP/Cy5.5-anti-CD45, FITC-Lin. Cells were subsequently fixed and permeabilized using Foxp3/Transcription Factor Buffer Set (eBioscience, America, 00−5523−00), followed by intracellular staining with APC-anti-Foxp3 or APC-anti-RORγt. Data were acquired on a BD FACS Canto II flow cytometer (BD Biosciences) and analyzed using FlowJo software (FlowJo, Ashland, OR, U.S.A.).

### 2.31 Evaluation of the engineered bacterial colonization and effect on colitis

To determine bacterial colonization and arabinose-inducible expression analysis, mice were pretreated with or without AVMN antibiotics for 5 d, followed by oral gavage with 5 × 10⁸ CFU of EcN (BAD−904) or control strains. To induce protein expression, 2% arabinose was provided in drinking water (changed every 48 h). Fecal samples were collected daily, and genomic DNA was extracted using Stool DNA Kit (Omega, America, D4015). Bacterial colonization was quantified via qPCR using Amuc_0904-specific primers (F: 5′-CACCTGAACACCGCCTACT−3′; R: 5′-GTTGAGCGGTTGATGTTCGT−3′) with the following cycling conditions: 95 °C for 10 min; 50 cycles of 95 °C for 30 s, 50 °C for 30 s, and 72 °C for 30 s. Colonization was expressed as relative abundance using the 2^−ΔΔCt^ method.

For biosafety assessment, mice received EcN (BAD−904) (5 × 10⁸ CFU) or controls via oral gavage every 48 h for 5 d. Major organs were collected for histopathological examination using H&E staining. Fecal samples were collected for microbial composition analysis via 16S rRNA sequencing.

To detect the preventive effect of EcN (BAD−904) on experimental colitis, mice received AVMN pretreatment for 5 d, followed by EcN (BAD−904) administration (5 × 10⁸ CFU every 48 h for 11 d) with 2% arabinose in drinking water. Colitis was induced from day 5 with 2% DSS water plus LPS (8 mg/kg, i.p.) for 6 d.

To evaluate the therapeutic effect of EcN (BAD−904) on experimental colitis, colitis was initiated with 2% DSS water for 7 d. From day 2 of DSS exposure, mice received EcN (BAD−904) (5 × 10⁸ CFU every 48 h) with 2% arabinose supplementation. Control groups received EcN (BAD-vector) or PBS.

### 2.32 Inhibitor treatment *in vivo*

Capmatinib was dissolved in DMSO (10 mg/mL stock) and diluted in a vehicle containing 40% PEG300, 5% Tween 80, and 45% saline to a working concentration of 1 mg/mL. Male C57BL/6J mice received daily oral gavage of capmatinib (10 mg/kg body weight) or vehicle control. In parallel, mice were administered EcN (BAD-vector) or EcN (BAD−904) (5 × 10⁸ CFU) every 48 h for 5 d. Bacterial expression was induced with 2% arabinose in drinking water.

### 2.33 Neutrophil depletion

After 48 h of 2% DSS exposure, mice were therapeutically treated with EcN (BAD−904) (5 × 10⁸ CFU every 48 h for 5 d) under 2% arabinose induction. Neutrophils were depleted via intraperitoneal injection of 0.2 mg anti‐mouse Ly6G antibody or isotype control at three time points: one day before DSS initiation, and on days 2 and 5 of DSS exposure.

### 2.34 Macrophage depletion

Following 48 h of 2% DSS exposure, mice received therapeutic oral gavage of EcN (BAD−904) (5 × 10⁸ CFU every 48 h for 5 d) with 2% arabinose induction. Macrophages were depleted by intravenous injection of clodronate liposomes or control liposomes (200 μL/mouse, Liposoma, Netherlands, CP005−005) 24 h before DSS initiation and on day 5 of colitis induction.

### 2.35 AOM/DSS-induced colitis-associated carcinogenesis

Male C57BL/6J mice received a single intraperitoneal injection of AOM (10 mg/kg, Sigma). After 7 d, mice were subjected to three cycles of 2% DSS drinking water (7 d) followed by 14 d of normal water. Throughout the 10-week experiment, mice received daily oral gavage of Amuc_0904 (10 μg/mouse) or EcN (BAD−904) (5 × 10⁸ CFU every 48 h). Body weight was monitored weekly. On day 70, colons were harvested, fixed in 4% PFA, and processed for histopathological evaluation.

### 2.36 Statistical analysis

Data are presented as mean ± standard deviation (SD). Statistical significance was determined using paired or unpaired Student’s t-test, one-way ANOVA, or two-way ANOVA. Statistical analyses were performed using Graphpad Prism 6.

## 3 Results

### 3.1 *A. muciniphila* isolated from colitis recovery alleviates DSS-induced colitis and enhances intestinal barrier

To evaluate the change and effect of human microbiota during colitis, C57BL/6J mice were pre-treated with an antibiotic cocktail (AVMN) for 7 d, followed by fecal microbiota transplantation (FMT) from healthy volunteers for 9 d, and then exposed to 1% DSS for 22 d (Figure S1A). The body weight and DAI scores changes of mice during the DSS treatment period were recorded (Figure S1B−C). We found that mice exhibited the acute inflammatory phase, characterized by increased DAI scores from 1 to 8 d, and began to recover with decreased DAI scores on the 8th d (Figure S1C). The gut microbiota and colon tissues of mice treated with DSS for 8th and 22nd d were collected and analyzed. According to DAI scores, colon length and histological morphology of mice treated with DSS for 8th d showed obvious symptoms of colitis and started to recover, whereas those of mice treated with DSS for 22nd d greatly recovered (Figure S1C−E). Furthermore, 16S rRNA sequencing of fecal samples collected from the DSS and control groups on the 8th and 22nd d, respectively, was carried out. Significant differences in the abundance and composition of fecal microbiota between mice in the acute inflammatory and remission phases were observed (Figure S1F−G). On the 8th d of the acute inflammatory phase, fecal microbiota abundance decreased significantly. Microbiota abundance increased by the 22nd d of the remission phase, with notably high levels of *Bacteroides*, *Akkermansia*, *Faecalibaculum*, and *Paraprevotella* genera (Figure S1H−I).

We further investigated the effects of enriched bacteria during the remission phase. Five strains isolated from human feces (*A. muciniphila*, *B. uniformis*, *B. intestinalis*, *B. oleiciplenus*, and *P. clara*) were respectively colonized in mice in addition to DSS treatment (Figure S1J). Among these strains, *A. muciniphila* exhibited the least weight loss, lowest DAI score, longest colon length, most intact colon tissue structure, and lowest histological score in mice (Figure S1K−R). Furthermore, we assessed the epithelial barrier function of mice colonized with *A. muciniphila* and treated with DSS. *A. muciniphila* significantly increased the expression of epithelial tight junction protein Occludin, the number of Ki67^+^ proliferating cells, the number of goblet cells, the expression of mucin protein (MUC2) secreted by goblet cells, and decreased the number of cleaved caspase−3^+^ apoptotic cells, compared with the control group (Figure S2A−E), indicating a protective effect on the intestinal barrier in DSS-induced colitis model. Moreover, we wondered whether *A. muciniphila* could directly regulate the intestinal barrier during homeostasis (Figure S2F). Immunohistochemical staining revealed that colonization with *A. muciniphila* significantly increased Occludin expression, Ki67^+^ proliferating cells, goblet cells, and MUC2 expression, with no effect on cleaved caspase−3^+^ apoptotic cells (Figure S2G−K).

Together, these results indicate that *A. muciniphila*, notably enriched during colitis remission, enhances intestinal barrier function by promoting epithelial integrity, proliferation, and goblet cell-mediated mucus production.

### 3.2 Amuc_0904 of *A. muciniphila* induces goblet cell characteristics

**Figure 1.**
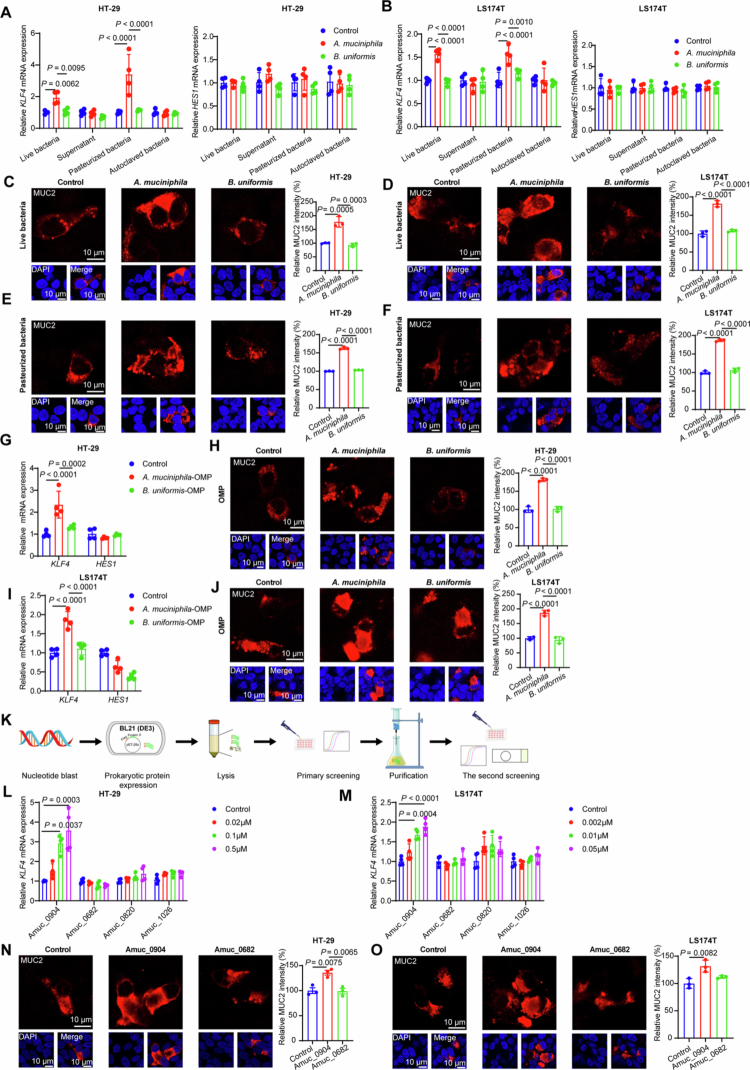
Amuc_0904 of *A. muciniphila* promotes goblet cell characteristics.(A) mRNA levels of *KLF4* and *HES1* in HT−29 cells treated with live, pasteurized, autoclaved *A. muciniphila*/*B. uniformis* (MOI = 2), or bacteria culture supernatant (20% v/v) for 72 h. PBS or blank bacteria culture medium was used as the control. (B) mRNA levels of *KLF4* and *HES1* in LS174T cells treated with live, pasteurized, autoclaved *A. muciniphila*/*B. uniformis* (MOI = 2), or bacteria culture supernatant (20% v/v) for 48 h. PBS or blank bacteria culture medium was used as the control. (C-D) Immunofluorescence analysis of MUC2 in HT−29 (C) or LS174T (D) treated with live *A. muciniphila* (MOI = 2, 72 h or 48 h). (E-F) Immunofluorescence analysis of MUC2 in HT−29 (E) or LS174T (F) treated with pasteurized *A. muciniphila* (MOI = 2, 72 h or 48 h). (G-J) HT−29 or LS174T were treated with outer membrane proteins of *A. muciniphila* (5 μg/mL, 72 h or 48 h). mRNA levels of *KLF4* and *HES1* in HT−29 (G) or LS174T (I), and immunofluorescence analysis of MUC2 in HT−29 (H) or LS174T (J). (K) Schematic of screening for *A. muciniphila* outer membrane protein that increases *KLF4* mRNA level. (L-O) HT−29 (0.5 μM, 72 h) or LS174T (0.05 μM, 48 h) cells were treated with Amuc_0904 or Amuc_0682. mRNA levels of *KLF4* in HT−29 cells (L) or LS174T cells (M), and immunofluorescence analysis of MUC2 in HT−29 cells (N) or LS174T cells (O). Scale bar: 10 μm. Data are the mean ± SD. *n* = 4 (A-B, G, I, L-M), *n* = 3 (C-F, H, J, N-O). One-way ANOVA (A-J, L-O).

Goblet cells are essential to protect intestinal barrier by producing mucin. We discovered that *A. muciniphila* effectively upregulated the *KLF4* mRNA level (the goblet cell differentiation marker) and MUC2 expression while not affecting the *HES1* mRNA level (the absorptive epithelial cell differentiation indicator) using colorectal cancer cell lines HT−29, LS174T, and Caco2 (Figure 1A−D and Figure S3A). Furthermore, we examined the effects of the pasteurized, autoclaved, proteinase K-treated *A. muciniphila*, and *A. muciniphila* culture supernatant. As proteinase K treatment abolished the effect of *A. muciniphila*, and pasteurized *A. muciniphila* still increased the *KLF4* mRNA level in HT−29, LS174T, and Caco2 cells (Figure 1A−F and Figure S3A−F), we speculated that the outer membrane proteins may be the effective components. As expected, stimulating cells with outer membrane proteins extracted from *A. muciniphila* increased the *KLF4* mRNA level and MUC2 expression in HT−29, LS174T, and Caco2 cells (Figure 1G−J and Figure S3G).

To identify the specific outer membrane proteins that promote *KLF4* mRNA and MUC2 expression, we searched for genes annotated to encode outer membrane proteins of *A. muciniphila*. These putative outer membrane protein-encoding genes were overexpressed in BL21 and the total protein solutions were used to stimulate HT−29 cells (Figure 1K). As shown in Figure S3H, we first identified 79 putative *A. muciniphila* outer membrane proteins, according to prior findings.^30^ After eliminating excessively long proteins and ribosomal proteins, 61 recombinant outer membrane proteins were chosen to be expressed. Unfortunately, only 26 recombinant outer membrane proteins were successfully expressed in the bacteria BL21, whereas 8 recombinant outer membrane proteins, including Amuc_0032, Amuc_0074, Amuc_0610, Amuc_0682, Amuc_0687, Amuc_0820, Amuc_0904, and Amuc_1026, were expressed in the supernatant, which could be purified (Figure S3I). The information of the 79 putative *A. muciniphila* outer membrane proteins was listed in Table S2. The total proteins from BL21 overexpressing Amuc_0820, Amuc_0904, and Amuc_1026 seemed to increase the *KLF4* mRNA level (Figure S3J), and we further obtained these purified proteins in addition to purified Amuc_0682 as a control. Purified Amuc_0904 protein, compared with other proteins, significantly increased the *KLF4* mRNA level in a concentration-dependent manner and stimulated MUC2 expression (Figure S3K and Figure 1L−O).

The whole-genome sequence of *A. muciniphila* isolated in this study was obtained and this bacteria strain was named *A. muciniphila* strain TMU (Figure S4A). A comparison of the genome sequences of *A. muciniphila* TMU with other *A. muciniphila* strains revealed that this strain belongs to the AmIII system and has a distant phylogenetic relationship with the standard strain *A. muciniphila* ATCC BAA−835, which belongs to the AmI system (Figure S4B). The sequence alignment of the gene encoding Amuc_0904 revealed its presence in most *A. muciniphila* strains, and the phylogenetic relationship of the Amuc_0904-encoding gene was consistent with that of the genome sequences, indicating its conservation (Figure S4C). To confirm the specific effect of Amuc_0904, we purified K12-AcnA protein (the homologous protein of Amuc_0904 in *E. coli* K12) and stimulated HT−29, LS174T, and Caco2 cell respectively, which had no effect on *KLF4* and MUC2 expression (Figure S4D−J).

Taken together, these results identify Amuc_0904 as a specific effector protein derived from *A. muciniphila* that directly promotes goblet cell differentiation.

### 3.3 Amuc_0904 affects Wnt signaling and oxidative phosphorylation (OXPHOS) pathways

**Figure 2.**
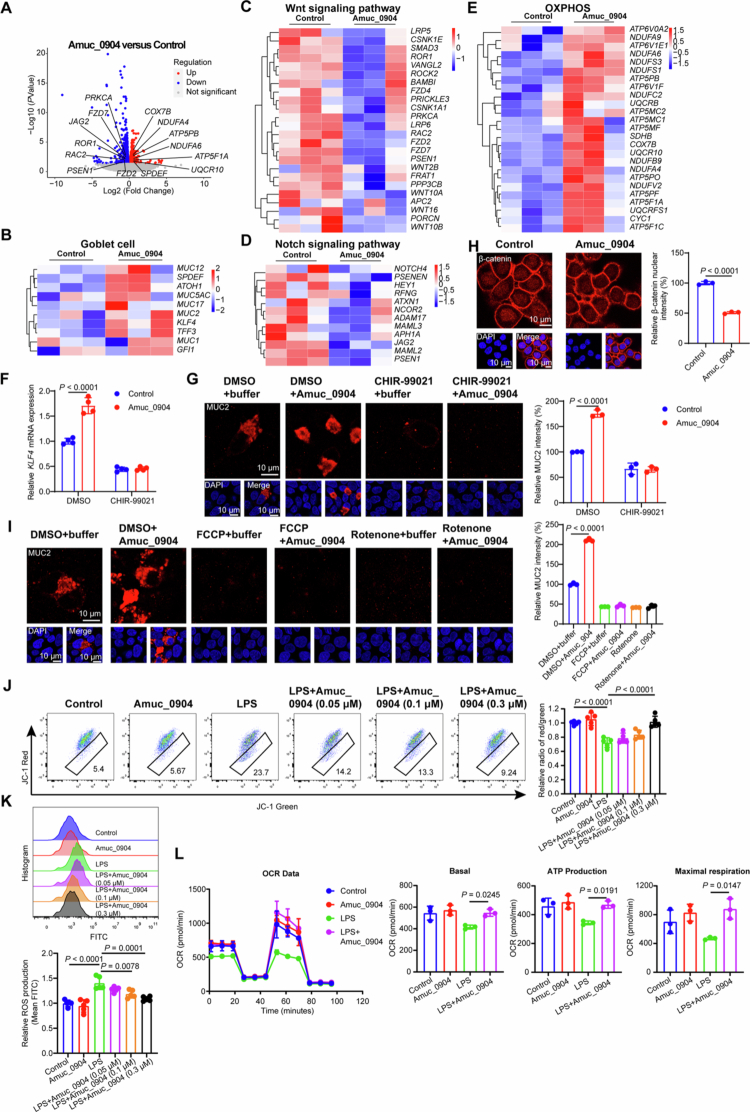
Amuc_0904 downregulates Wnt signaling pathway and upregulates OXPHOS pathway.(A-E) RNA-seq analysis of HT−29 cells stimulated with buffer or Amuc_0904 (0.5 μM, 72 h). (A) Volcano plot showing the upregulated and downregulated genes. (B-E) Heatmap of goblet cell signature genes (B), Wnt signaling pathway associated genes (C), Notch signaling pathway associated genes (D), and OXPHOS associated genes (E).(F-G) HT−29 cells were treated with Wnt signaling pathway activator CHIR−99021 (10 μM, 72 h) or DMSO in combination with or without Amuc_0904 (0.5 μM, 72 h). mRNA levels of *KLF4* (F) or immunofluorescence analysis of MUC2 (G). (H) Immunofluorescence analysis of *β*-catenin in HT−29 cells treated with buffer or Amuc_0904 (0.5 μM, 72 h). (I) Immunofluorescence analysis of MUC2 in HT−29 cells treated with respiratory chain inhibitors FCCP (20 μg/mL, 72 h) and Rotenone (20 μg/mL, 72 h). (J-L) Caco2 cells were treated with LPS (1 μg/mL, 36 h) in combination with or without Amuc_0904 (0.3 μM, 12 h). Analysis of mitochondrial membrane potential (J), the cellular ROS levels (K), and the cellular OCR levels (L) in the indicated Caco2 cells. Scale bar: 10 μm. Data are the mean ± SD. *n* = 3 (A-E, G-I, L), *n* = 4 (F). *n* = 5 (J-K). Two-way ANOVA (F-G), one-way ANOVA (I-L) or unpaired t tests (H).

To elucidate the mechanism by which Amuc_0904 increases *KLF4* mRNA and MUC2 expression, RNA-seq was performed to examine the gene expression profiles of HT−29 cells treated with buffer or Amuc_0904. It is revealed that 565 genes were upregulated and 579 genes were downregulated in the Amuc_0904 group (*p* < 0.05 and fold change > 1.5) (Figure 2A). Goblet cell-related genes such as *MUC2*, *TFF3, MUC12*, *MUC1*, *MUC5AC*, *SPDEF*, *ATOH1* and *KLF4* were upregulated in the Amuc_0904-treated group (Figure 2B). Heatmap and KEGG enrichment analysis revealed the downregulation of Wnt and Notch signaling pathways in the Amuc_0904 group (Figure 2C−D and Figure S5A), whereas oxidative phosphorylation (OXPHOS) metabolic pathway was significantly upregulated (Figure 2E and Figure S5B).

Wnt and Notch signaling pathways are involved in intestinal epithelial cell differentiation, and we investigated their roles in Amuc_0904-induced regulation of *KLF4* mRNA and MUC2 expression. HT−29 cells were treated with the Wnt signaling activator (CHIR−99021) and the Notch signaling inhibitor (DAPT), respectively. CHIR−99021 treatment inhibited the effect of Amuc_0904, while DAPT had no effect (Figure 2F−G and Figure S5C−D). Moreover, immunofluorescence staining revealed that Amuc_0904 decreased the nuclear intensity of *β*-catenin, a target protein involved in Wnt signaling pathway (Figure 2H).

To investigate the role of OXPHOS on Amuc_0904-induced MUC2 expression, we treated HT−29 cells with respiratory chain inhibitors FCCP and Rotenone, and the MUC2 upregulation by Amuc_0904 was inhibited (Figure 2I). Given that a high OXPHOS level indicates enhanced mitochondrial function and mitochondrial oxidative energy metabolism is critical in reducing oxidative stress during inflammation, we examined ROS production and mitochondrial membrane potential under LPS and Amuc_0904 treatment. Flow cytometry and fluorescence staining showed that Amuc_0904 increased the ratio of mitochondrial aggregates to monomers, reduced cellular ROS levels, and elevated mitochondrial membrane potential in epithelia, indicating that Amuc_0904 improves mitochondrial function (Figure 2J-K and Figure S5E−F). Additionally, Amuc_0904 restored basal/maximum mitochondrial respiration and ATP-producing capacity decreased by LPS (Figure 2L).

Collectively, these results demonstrate that Amuc_0904 promotes goblet cell characteristics by downregulating Wnt signaling pathway and activating OXPHOS pathway, while Amuc_0904-induced OXPHOS also improves mitochondrial function in epithelia.

### 3.4 Amuc_0904 interacts with MET to affect Wnt signaling and OXPHOS pathways

**Figure 3.**
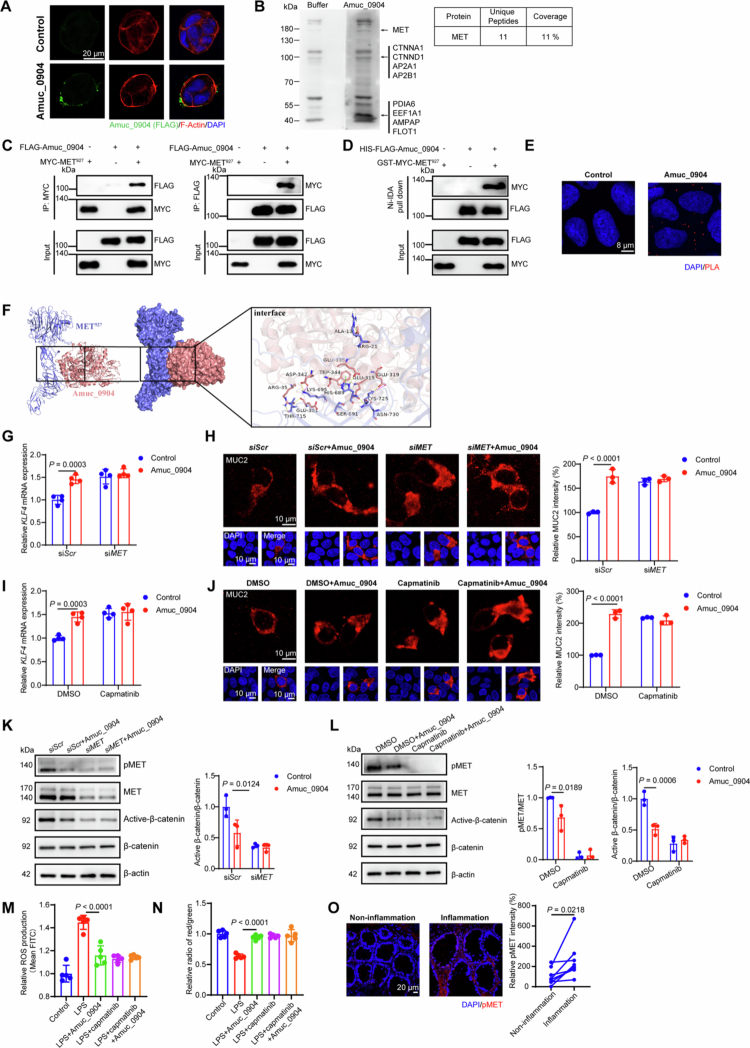
MET is responsible for Amuc_0904-induced goblet cell differentiation. (A-B) Caco2 cells were treated with buffer or Amuc_0904 (0.3 μM, 12 h). Immunofluorescence analysis of FLAG-Amuc_0904 and F-Actin (A), Far-Western combined with mass spectrometry analysis of Amuc_0904 interacted proteins (B). (C) Co-immunoprecipitation analysis of the interaction between the purified MYC-MET^927^ and FLAG-Amuc_0904 proteins. (D) Ni-IDA pull-down assay of the interaction between the purified GST-MYC-MET^927^ and HIS-FLAG-Amuc_0904 proteins. (E) Duolink PLA analysis of direct interaction between MET and FLAG-Amuc_0904 in Caco2 cells treated with buffer or Amuc_0904 (0.3 μM, 12 h). (F) Docking Amuc_0904 into the human MET. (G-L) HT−29 cells were treated with si*MET* transfection or capmatinib (10 nM, 72 h) in combination with or without Amuc_0904 (0.5 μM, 72 h). mRNA levels of *KLF4* (G, I) and immunofluorescence analysis of MUC2 (H, J). (K-L) Western blots of *p*-MET and active-*β*-catenin protein levels in the indicated HT−29 cells. The signal densities of *p*-MET were normalized to those of total MET. The signal densities of active-*β*-catenin were normalized to those of total *β*-catenin. (M-N) Caco2 cells were pretreated with LPS (1 μg/mL, 36 h) in combination with or without Amuc_0904 (0.3 μM) and capmatinib (10 nM) for 12 h. Cellular ROS (M) and mitochondrial membrane potential (N). (O) Immunohistochemical analysis of *p*-MET in colon tissues from human colitis samples. Scale bar: 8 μm, 10 μm or 20 μm. Data are the mean ± SD. *n =* 4 (G, I), *n =* 3 (H, J-L), *n* = 5 (M-N) or *n =* 8 (O). Two-way ANOVA (G-L), one-way ANOVA (M-N) or paired t tests (O).

The immunofluorescence staining revealed that Amuc_0904 co-localized with F-Actin in Caco2 cells, indicating that Amuc_0904 interacts with cell membrane proteins to influence cell function (Figure 3A). We further employed far-western blotting and MS to identify the Amuc_0904-targeted proteins on the cell membrane (Figure 3B). We noted the protein MET, which is a tyrosine kinase receptor and is involved in cell proliferation and differentiation through activating multiple signaling pathways including Wnt/β-catenin signaling. To validate the direct interaction between Amuc_0904 and MET, MYC-MET^927^ (the extracellular domain of MET) was purified and immunoprecipitated with FLAG-Amuc_0904. MYC-MET^927^ was immunoprecipitated with FLAG-Amuc_0904 and vice versa (Figure S5G and Figure 3C). Ni-IDA pull-down experiments and Duolink proximity ligation assay (PLA) further verified the direct interaction between FLAG-Amuc_0904 and MET (Figure 3D−E). Moreover, molecular docking results revealed that Amuc_0904 binds to the Ig2 domain of MET (Figure 3F). To validate the specific binding between Amuc_0904 and the MET receptor, we firstly compared the amino acid sequence of Amuc_0904 at MET binding sites with that of *E. coli* K12 AcnA. There were three different amino acids at binding sites which may promoted the different MET binding of Amuc_0904 compared with *E. coli* K12 AcnA (Figure S5H). Then, *E. coli* K12 AcnA was also docked into the MET receptor. The result revealed that the domain of MET binding Amuc_0904 is different from that of *E. coli* K12 AcnA and Amuc_0904 has a stronger interaction with MET, compared with *E. coli* K12 AcnA (Figure S5I).

We further examined the role of MET in Amuc_0904-induced *KLF4* mRNA and MUC2 expression. Amuc_0904 abolished the upregulation of *KLF4* mRNA and MUC2 expression in HT−29 cells transfected with si*MET* (Figure 3G−H). The MET inhibitor capmatinib, which inhibits MET phosphorylation, has a similar effect to si*MET* transfection (Figure 3I−J). To further assess whether the MET binding capacity and domain of Amuc_0904 are critical for MET dephosphorylation, we measured the levels of *p*-MET in HT−29 cells treated with Amuc_0904 or *E. coli* K12 AcnA. The result showed that MET was indeed dephosphorylated by Amuc_0904 but not by *E. coli* K12 AcnA (Figure S5J). Western blot revealed that Amuc_0904 reduced *p*-MET levels without affecting total MET expression, as well as decreased active-*β*-catenin expression without affecting total *β*-catenin levels (Figure 3K−L). Of note, si*MET* transfection and capmatinib treatment reduced active-*β*-catenin/β-catenin levels, indicating that inhibiting MET activation suppresses Wnt signaling pathway (Figure 3K−L). Inhibiting MET phosphorylation also abolished Amuc_0904-restored cellular ROS levels and the ratio of mitochondrial aggregates to monomers under LPS simulation, according to flow cytometry analysis (Figure 3M−N). Consistent with the *in vitro* data, the phosphorylation of MET was higher in inflamed colon tissues from colitis samples than in adjacent non-inflamed colon tissue (Figure 3O).

Taken together, these findings indicate that Amuc_0904 specifically binds to the MET receptor, inhibits MET phosphorylation, and consequently attenuates Wnt/β-catenin signaling while enhancing mitochondrial function.

### 3.5 Amuc_0904 promotes goblet cell differentiation in colonic organoids via the MET-Wnt-OXPHOS axis

**Figure 4.**
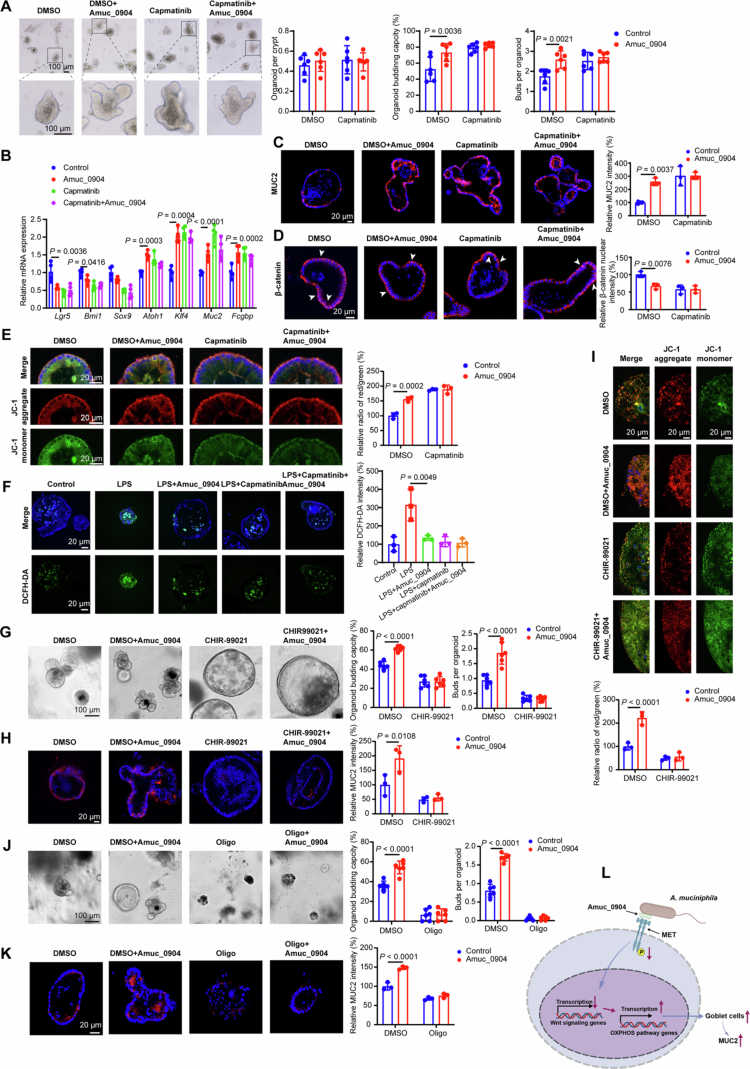
Amuc_0904 promotes goblet cell differentiation and enhances mitochondrial function in colonic organoids. (A-E) Colonic organoids isolated from WT mice were treated with capmatinib (20 nM) and Amuc_0904 (0.5 μM) for 4 d. (A) Bright-field images of colonic organoids. (B) mRNA levels of stem cell-related genes (*Lgr5*, *Bmi1*, and *Sox9*) and goblet cell-related genes (*Atoh1*, *Klf4*, *Muc2*, and *Fcgbp*). (C-D) Immunofluorescence analysis of MUC2 (C) and *β*-catenin (D). (E) Mitochondrial membrane potential in colonic organoids. (F) Cellular ROS in colonic organoids treated with LPS (10 μg/mL) in combination with or without Amuc_0904 (0.5 μM) and capmatinib (20 nM) for 72 h. (G-I) Colonic organoids were treated with CHIR−99021 (10 μM) and Amuc_0904 (0.5 μM) for 72 h. (G) Bright-field images of colonic organoids. (H) Immunofluorescence analysis of MUC2. (I) Mitochondrial membrane potential in colonic organoids. (J-K) Colonic organoids were treated with Oligomycin A (Oligo, 5 μM) and Amuc_0904 (0.5 μM) for 72 h. (J) Bright-field images of colonic organoids. (K) Immunofluorescence analysis of MUC2. (L) Schematic summarizing the mechanisms of Amuc_0904 promotes goblet differentiation. Schematic diagrams were generated by BioRender.com. Scale bar: 20 μm or 100 μm. *n =* 6 (A, G, J), *n =* 4 (B), *n* = 3 (C-F, H-I, K). Data are the mean ± SD. Two-way ANOVA.

To further validate the effect of Amuc_0904 on goblet cell differentiation, colonic crypts isolated from WT mice were used. Amuc_0904 treatment significantly increased organoid budding capacity and bud counts, while having no apparent effect on organoid formation capacity (Figure 4A). In addition, Amuc_0904 reduced mRNA levels of stem cell-related genes *Lgr5*, *Bmi1*, and *Sox9*, promoted the mRNA expression of goblet cell-related genes *Atoh1*, *Klf4*, *Muc2*, *Fcgbp*, and increased MUC2 expression, which indicates that Amuc_0904 promotes stem cell differentiation into goblet cells (Figure 4B−C). These results suggest that Amuc_0904 drives the differentiation of intestinal stem cells toward a goblet cell lineage.

Furthermore, Amuc_0904 treatment suppressed nuclear translocation of *β*-catenin, enhanced mitochondrial membrane potential, and attenuated LPS-induced reactive oxygen species (ROS) production (Figure 4D−F), indicating modulation of Wnt signaling and mitochondrial function. The effects of Amuc_0904 on goblet cell differentiation, Wnt activity, and mitochondrial function were abolished by capmatinib (a MET inhibitor), supporting the involvement of MET signaling (Figure 4A–F).

To determine whether sustained Wnt activation can rescue the effects of Amuc_0904 in colonic organoids, we administered CHIR−99021, a Wnt agonist, alongside Amuc_0904 in colonic organoids. The results demonstrated that Wnt activation effectively counteracted Amuc_0904-induced goblet cell differentiation and the enhancement of OXPHOS (Figure 4G−I).

Given OXPHOS is critical for goblet cell differentiation, we next investigated the essential role of OXPHOS in Amuc_0904-mediated goblet cell differentiation. We treated colonic organoids with Amuc_0904 in combination with oligomycin, a specific OXPHOS inhibitor. Oligomycin treatment markedly attenuated Amuc_0904-induced goblet cell differentiation, as evidenced by decreased organoid budding capacity and diminished MUC2 expression (Figure 4J−K). Collectively, these findings indicate that Amuc_0904 mediated goblet cell differentiation in colonic organoids through Met-Wnt-OXPHOS axis.

Collectively, these results demonstrate that Amuc_0904 induces goblet cell differentiation in colonic organoids through a MET-Wnt-OXPHOS signaling axis (Figure 4L).

### 3.6 Amuc_0904 protects mice against colitis and colitis-associated colorectal cancer

**Figure 5.**
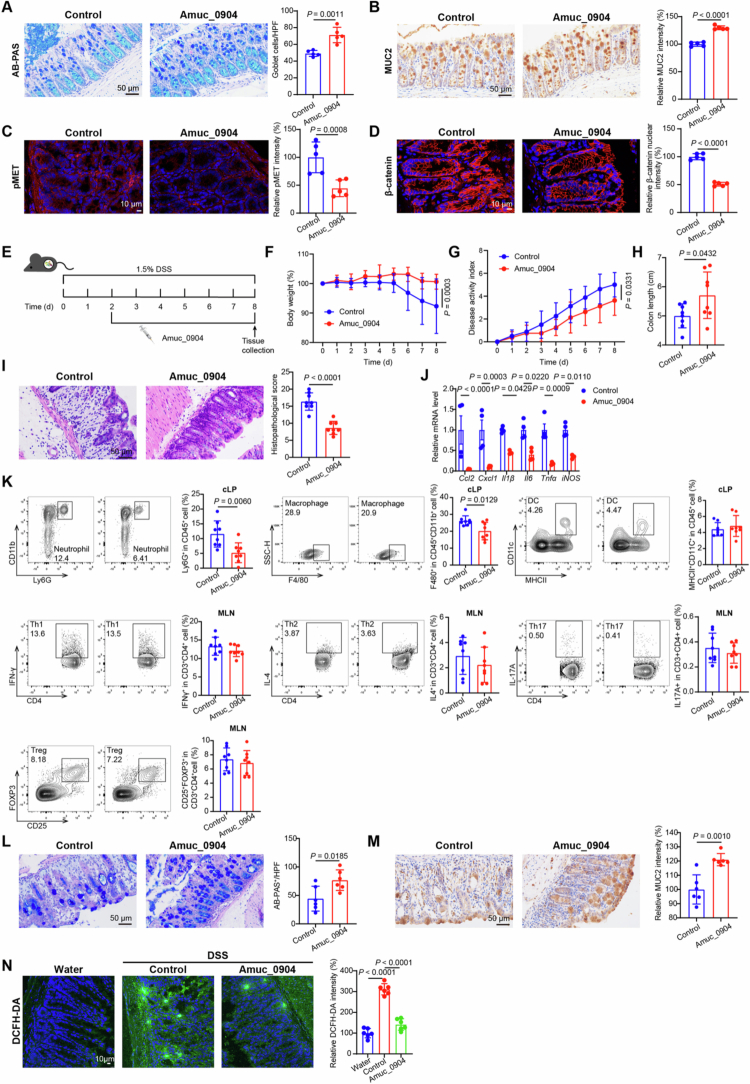
Amuc_0904 enhances goblet cell numbers and alleviates colitis.(A-D) Mice were administered with Amuc_0904 (10 μg) for 5 d. AB/PAS staining (A), immunohistochemical analysis of MUC2 (B), immunofluorescence analysis of *p*-MET (C) or *β*-catenin (D) in colon tissues. (E-N) After 2 d of 1.5% DSS treatment, the C57BL/6J mice were oral administrated Amuc_0904 (10 μg) for 6 d. (E) Schematic diagram for the therapeutic model. Analysis of body weight (F), DAI scores (G), colon length (H) and histological scores (I) of the indicated mice. (J) mRNA expression levels of proinflammatory cytokines in the colons. (K) Representative flow cytometry analysis of the indicated cells (left) and percentage (right) of indicated cells in cLP and MLN from indicated mice. AB/PAS staining (L), and immunohistochemical staining analysis of MUC2 (M) in colon tissues. (N) The ROS levels in the colons. Scale bar: 10 μm or 50 μm. Data are the mean ± SD. *n =* 5 (A-D), *n* = 8 (F-I, K), *n* = 4 (J), *n* = 6 (L-N). Two-way ANOVA (F-G, J), one-way ANOVA (N) or unpaired t tests (A-D, H-I, K-M).

To further validate the role of Amuc_0904 *in vivo*, we examined whether Amuc_0904 protein regulated goblet cell differentiation under normal conditions. Amuc_0904 protein was found to promote the increased goblet cell number and MUC2 expression in colon tissue (Figure 5A−B). Furthermore, the level of *p*-MET expression and *β*-catenin nuclear expression were both decreased in these mice treated with Amuc_0904 protein (Figure 5C−D), indicating that Amuc_0904 protein inhibits Wnt signaling pathway and MET phosphorylation in mice.

Next, the protective effects of Amuc_0904 protein on DSS-induced colitis were determined in the therapeutic model (Figure 5E). Amuc_0904 protein promoted recovery of body weight loss, disease activity scores, shortened colon length, and histological damage (Figure 5F−I). Moreover, Amuc_0904 protein treatment significantly reduced the expression of inflammatory factors in mouse intestinal tissues, including *Ccl2, Cxcl1, Il1β, Il6, Tnfα*, and *iNOS* (Figure 5J). Decreased percentage of neutrophils and macrophages in the colon were observed in Amuc_0904 protein-treated mice (Figure S6A-B and Figure 5K). The number of goblet cells and MUC2 expression were also increased in colon tissues from the Amuc_0904 protein group (Figure 5L−M). In addition, the effects of Amuc_0904 protein in colitis prevention model were also consistent with those in colitis therapeutic model (Figure S6C−K). Meanwhile, ROS production was also decreased in colonic tissues of Amuc_0904 protein-treated mice, demonstrating that Amuc_0904 protein promotes mitochondrial function and reduces oxidative stress induced by colitis (Figure 5N).

Since colitis with long-term chronic inflammation increases the incidence of colitis-associated colorectal cancer (CAC), we sought to find out whether the protective effects of Amuc_0904 protein on colitis could suppress the progression of CAC. Mice were treated with azoxymethane (AOM) and DSS to induce colitis-associated colorectal tumors (Figure S7A). Remarkably, Amuc_0904 protein-treated mice lost significantly less body weight and had fewer tumors of smaller size than the control group (Figure S7B−C). Consistently, Amuc_0904 protein-treated mice exhibited benign adenomas with fewer Ki67^+^ proliferating cells in the colonic tissues, whereas the control group developed malignant tumors with more Ki67^+^ proliferating cells (Figure S7D−E).

Together, these data suggest that Amuc_0904 protein administration effectively promotes goblet cell differentiation, alleviates colitis, and inhibits tumorigenesis.

### 3.7 Engineered EcN expressing Amuc_0904 increases goblet cells and mucin in mice through MET

**Figure 6.**
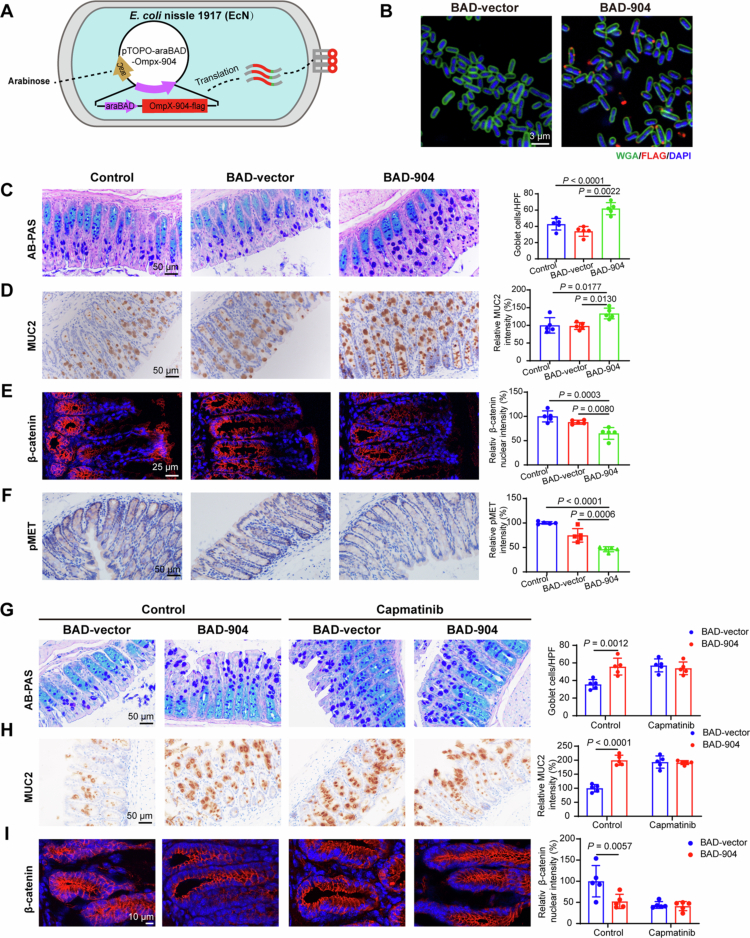
Engineered EcN (BAD−904) enhances goblet cell numbers and mucin secretion by MET.(A) Construction of engineered EcN (BAD−904) with Amuc_0904 overexpression induced by arabinose. (B) Immunofluorescence analysis of FLAG- Amuc_0904 and WGA in engineered EcN (BAD−904). (C-F) Mice were administered with 5 × 10^8^ CFU of EcN (BAD-vector) or EcN (BAD−904) with 2% arabinose in drinking water for one week. PBS was used as the control. AB/PAS staining (C), immunohistochemical analysis of MUC2 (D), immunofluorescence analysis of *β*-catenin (E), and immunohistochemical analysis of *p*-MET (F) in colon tissues. (G-I) Mice were administered with 5 × 10^8^ CFU of EcN (BAD-vector) or EcN (BAD−904) with 2% arabinose in drinking water, with or without capmatinib (10 mg/kg) treatment for 5 d. The solvent was used as the control. AB/PAS staining (G), immunohistochemical analysis of MUC2 (H), immunofluorescence analysis of *β*-catenin (I) in colon tissues. Scale bar: 3 μm, 10 μm, 25 μm or 50 μm. Data are the mean ± SD. *n* = 5 (C-I). Two-way ANOVA (G-I) or one-way ANOVA (C-F).

As genetically engineered probiotics that could be delivered effectively and continuously produce therapeutics have been reported to treat colitis, we developed the recombinant plasmid pTOPO-araBAD-Ompx−904 (BAD−904), for Amuc_0904 expression as an outer membrane protein tagged with FLAG and controlled by an arabinose-inducible promoter (Figure 6A). Amuc_0904 in EcN with BAD−904 was detected on the bacterial surface under arabinose induction (Figure 6B). EcN (BAD−904) successfully colonized the intestinal tract and produced functional FLAG-tagged Amuc_0904 protein, with or without AVMN antibiotic pretreatment (Figure S7F−I). Furthermore, EcN (BAD−904) administered orally for one week significantly increased goblet cells and MUC2 expression in mouse colon tissues (Figure 6C−D). Additionally, EcN (BAD−904) administration decreased epithelial *β*-catenin nuclear expression, indicating that the engineered bacteria inhibit Wnt signaling pathway in mice (Figure 6E). We then investigated whether the effect of EcN (BAD−904) *in vivo* is dependent on MET phosphorylation. As expected, decreased MET phosphorylation was observed in colon tissues from EcN (BAD−904)-treated mice (Figure 6F). Additionally, mice treated with capmatinib showed no difference in goblet cells, MUC2 levels and *β*-catenin nuclear expression between the EcN (BAD-vector) and EcN (BAD−904) groups (Figure 6G−I).

Furthermore, we assessed the biosafety of the engineered bacteria administered via gavage for one week. The histopathology of colon, liver, spleen, lung, and kidney tissues from mice treated with EcN (BAD−904) exhibited no obvious difference from that of control mice (Figure S7J). In addition, 16 s rDNA sequencing of fecal samples revealed that EcN (BAD−904) colonization did not alter the gut microbiota (Figure S7K−N).

Together, these results demonstrate that EcN (BAD−904) inhibits Wnt signaling pathway and goblet cell differentiation *in vivo* via MET.

### 3.8 EcN (BAD−904) protects mice against colitis and colitis-associated colorectal cancer

**Figure 7.**
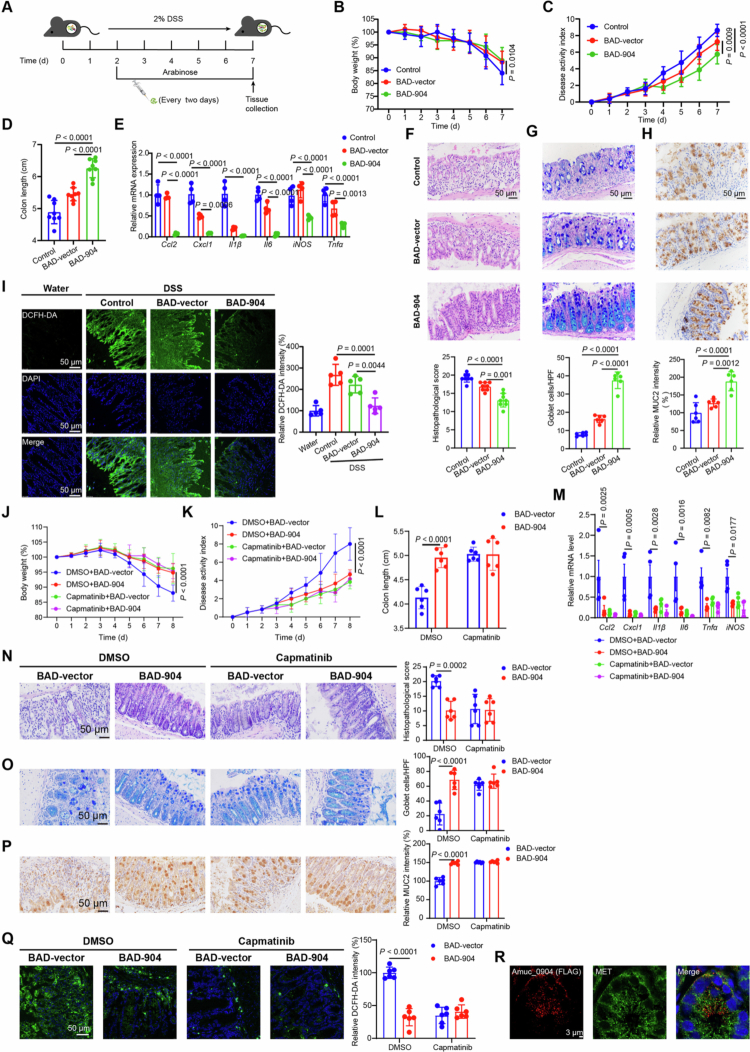
EcN (BAD−904) alleviated colitis in mice through MET. (A-I) After 2 d of 2% DSS treatment, the C57BL/6J mice were oral administrated EcN (BAD−904) every two days for 5 consecutive days with 2% arabinose in drinking water. (A) Schematic diagram for the therapeutic model. Analysis of body weight (B), DAI scores (C), and colon length (D) of the indicated mice. (E) mRNA expression levels of proinflammatory cytokines in the colons. (F-H) Colon tissues were collected from the indicated mice of the therapeutic model and were used to perform H&E and immunohistochemical staining analysis. H&E staining and histological scores (F), AB/PAS staining (G), and immunohistochemical staining analysis of MUC2 (H) in the colon tissue. (I) The ROS levels in the colons. (J-R) After 2 d of 1.5% DSS treatment, mice were administered with 5 × 10^8^ CFU of EcN (BAD-vector) or EcN (BAD−904) with 2% arabinose in drinking water, with or without capmatinib (10 mg/kg) treatment for 6 d. Analysis of body weight (J), DAI scores (K), and colon length (L) of the indicated mice. (M) mRNA expression levels of proinflammatory cytokines in the colons. H&E staining and histological (N), AB/PAS staining (O), and immunohistochemical staining analysis of MUC2 (P) in colon tissues. (Q) The ROS levels in the colons. (R) Immunofluorescence staining to evaluate co-localization of Amuc_0904 (FLAG, red) and MET (green) in the colon of mice treated with EcN (BAD−904). Scale bar: 50 μm or 3 μm. Data are the mean ± SD. *n =* 8 (B-D, F), *n =* 6 (G-H, J-L, N-Q), *n* = 5 (I), *n =* 4 (E, M). Two-way ANOVA (B-C, E, J-Q) or one-way ANOVA (D, F-I).

The role of EcN (BAD−904) in the protective phenotype was next determined *in vivo* using DSS-induced colitis therapeutic model. Similar to Amuc_0904, EcN (BAD−904) treatment promoted recovery of body weight loss, disease activity scores, shortening of the colon, and intestinal inflammation in DSS-induced colitis prevention model (Figure 7A−E). The proportion of neutrophils and macrophages in the colon was significantly reduced in EcN (BAD−904)-pretreated mice (Figure S8A). Furthermore, more intact morphology, increased goblet cell numbers, and increased MUC2 expression were observed in colon tissues from the EcN (BAD−904) group (Figure 7F−H). Meanwhile, ROS production was reduced in colonic tissues of EcN (BAD−904)-treated mice, demonstrating that EcN (BAD−904) improves mitochondrial function and reduces oxidative stress caused by colitis (Figure 7I).

We further established the DSS-induced colitis model to assess the prevention efficacy of EcN (BAD−904) (Figure S8B). Consistent with the results observed in the prevention model, EcN (BAD−904) showed protective effects according to disease phenotype (Figure S8C−J).

To further determine whether the effect of EcN (BAD−904) *in vivo* is dependent on MET phosphorylation, capmatinib treatment was performed in mice administered with EcN (BAD-vector) or EcN (BAD−904) under colitis conditions. The results showed that capmatinib could relieve colitis in mice, as manifested by reduced weight loss, decreased DAI scores, colon length, expression of tissue inflammatory factors, HE scores, ROS production, and immune cell infiltration, as well as an increased number of goblet cells and MUC2 expression (Figure 7J−Q and Figure S9A). Moreover, capmatinib treatment eliminated the difference between EcN (BAD−904) and EcN (BAD-vector), suggesting that MET phosphorylation was responsible for the protective effects of EcN (BAD−904) on colitis. In addition, immunofluorescent staining analysis revealed that Amuc_0904 (FLAG) co-localized with MET in mouse colon tissues (Figure 7R).

Since previous studies suggest that MET is widely expressed in colon tissue, including epithelial cells and immune cells,^21^^,^^31^ we sought to evaluate the potential contribution of immune cells to the protective effects of EcN (BAD−904). We analyzed immune cell populations in the colonic lamina propria (cLP) and mesenteric lymph nodes (MLNs) of mice treated with EcN (BAD−904) under steady-state conditions. Flow cytometric analysis revealed that the frequencies of major immune subsets-including macrophages, neutrophils, DCs, Th1/Th2/Th17 cells, Treg, and ILC3 cells in cLP, as well as T helper subsets and Treg in MLN-were comparable between EcN (BAD−904)-treated and control groups (Figure S9B−C). Furthermore, the proportions of CD45⁺ immune cells and CD3⁺ T cells in the intraepithelial compartment were unaltered by EcN (BAD−904) treatment (Figure S9D−E). These results indicate that EcN (BAD−904) does not broadly disrupt immune homeostasis, supporting that its protective effects may operate primarily through epithelial mechanisms. We previously found that infiltrating lymphocytes including macrophages and neutrophils were obviously decreased in mice with EcN (BAD−904) treatment after DSS administration, which may be the result of colitis remission but not the inducement of colitis remission (Figure S8A). To further exclude the role of neutrophils and macrophages in the protective effects of EcN (BAD−904), we used Ly6G antibody and clodronate liposomes (CLP) to induce neutrophil and macrophage depletion in mice with colitis. The results showed that EcN (BAD−904) treatment with neutrophil and macrophage depletion still had the effect of alleviating colitis, implying that EcN (BAD−904) treatment alleviated colitis independently of immune cells (Figure S10A-H and Figure S11A−H). Furthermore, EcN (BAD−904) co-localized with Epcam (epithelial cell marker) rather than CD45 (immune cell marker) were observed in mice colonic tissue (Figure S11I−J), demonstrating that EcN (BAD−904) targets epithelial cells in the gut. Collectively, our results suggest that immune cells are dispensable for the protective effects of EcN (BAD−904).

Meanwhile, EcN (BAD−904) administration effectively inhibited tumorigenesis in CAC mouse model, characterized by reduced decrease in body weight, fewer tumor numbers, smaller tumor volumes, and significantly decreased Ki−67 expression (Figure S12A−E). Furthermore, we noted that the phosphorylation levels of MET were dramatically decreased in the colon tissues of EcN (BAD−904)-treated mice (Figure S12F). We further observed a significant increase in MET phosphorylation in tumor tissues compared with matched para-carcinoma tissues from patients with colorectal cancer (Figure S12G).

Overall, these data suggest that EcN (BAD−904) is effective both as a preventative and therapeutic treatment for colitis, as well as attenuates colitis-induced colorectal tumorigenesis.

## 4 Discussion

*A. muciniphila*, one of the most abundant members of the gut microbiota, has emerged as a promising probiotic agent due to its role in regulating host health.^11^ Previous research revealed that *A. muciniphila* may regulate intestinal homeostasis by activating the ALPK1/TIFA/TRAF6 axis.^32^ A recent study found that *A. muciniphila*’s secreted protein Amuc_1409 improved intestinal stem cell regeneration by activating Wnt/β-catenin signaling.^33^ Some studies identified that pasteurized *A. muciniphila* and the OM protein Amuc_1100 reduced tumorigenesis and obesity development.^34^^,^^35^ However, the role and mechanism of *A. muciniphila* in goblet cells are unclear. Here, we determined that *A. muciniphila* contributed to intestinal homeostasis via Amuc_0904 (a previously uncharacterized outer membrane protein)-induced goblet cell differentiation. As an exclusive mucin-degrading bacterium, *A. muciniphila*’s effect on mucin-secreting was clarified in detail for the first time, which may benefit its survival in the intestine in addition to maintaining homeostasis (Figure S13).

MET has been identified as a receptor for a few bacterial surface proteins including *C. albicans* Hyr1 and *L*. *monocytogenes* InlB, which mediate the endocytosis of these organisms​​​​​​.^36^^,^^37^ Furthermore, *H. pylori* CagA targets MET intracellularly, promoting epithelial cell motility and invasiveness.^38^ In this study, we discovered that *A. muciniphila* Amuc_0904 interacted with MET and inhibited its phosphorylation. The precise mechanism by which Amuc_0904 reduced MET phosphorylation requires further investigation.

Different bacterial strains, even the same species, have distinct genomic homology leading to discrepant function.^39^^,^^40^ *A. muciniphila* strains are classified into four phylogroups (AmI, AmII, AmIII, and AmIV), with functional annotation indicating their distinct metabolic and functional characteristics.^41^ *A. muciniphila* strain TMU isolated in this study belongs to the AmIII phylogroup, which is different from *A. muciniphila* ATCC BAA−835 (AmI), mostly studied in published reports.^2^^,^^10^^,^^18^ The peptide Amuc_0904 shares 52% identity with *E. coli* K12 AcnA*,* which is induced by iron and oxidative stress and has a protective role against oxidative stress.^42^ However, the MET binding capacity and domain of Amuc_0904 were different from those of *E. coli* K12 AcnA, indicating that *E. coli* AcnA failed to function similarly to Amuc_0904. The unique way that Amuc_0904 binds to MET may play a special role in regulating goblet cell differentiation.

Although administration of engineered EcN (BAD−904) did not alter immune cell populations under steady-state conditions, it significantly ameliorated colitis pathology. This therapeutic effect was accompanied by reduced pro-inflammatory cytokines and diminished infiltration of neutrophils and macrophages. Depletion experiments suggested that these innate immune cells are not strictly necessary for the protective effect of EcN (BAD−904) (Figure S10−S11). Instead, the observed immunomodulation is likely secondary to Amuc_0904-induced enhancement of epithelial barrier function and goblet cell differentiation, rather than direct immune regulation. By improving mucosal integrity and promoting epithelial repair, EcN (BAD−904) may reduce microbial translocation and subsequent immune activation, thereby attenuating overall inflammation.

Notably, several bioactive molecules derived from *A. muciniphila*-such as Amuc_1100 and the *β*-carboline alkaloid harmaline-have been shown to inhibit NF-κB-mediated inflammation.^19^^,^^43^ Although our study primarily focused on barrier function enhancement, we cannot exclude the possibility that Amuc_0904 may also directly modulate epithelial inflammatory signaling pathways, potentially via NF-κB. Future studies should explore whether Amuc_0904 influences NF-κB or other immunoregulatory pathways.

Our results indicate that Amuc_0904 enhances mitochondrial function, as shown by increased membrane potential and reduced ROS levels, supporting the growing evidence that OXPHOS is essential for goblet cell differentiation^44^. A key mechanistic insight arising from this study is how Amuc_0904-mediated inhibition of MET/Wnt signaling leads to enhanced mitochondrial OXPHOS. We propose that suppression of Wnt signaling triggers a metabolic shift from glycolysis to oxidative phosphorylation, supplying the energy and biosynthetic precursors required for goblet cell maturation and mucin production. This transition may involve downregulation of downstream Wnt targets such as c-Myc, a known regulator of glycol metabolism.^45^^,^^46^ Reduced c-Myc expression could alleviate its suppression of mitochondrial biogenesis and function, thereby enhancing OXPHOS activity to meet the high energy demands of secretory differentiation. Future studies directly assessing metabolic flux and c-Myc expression in response to Amuc_0904 will be crucial to validate this pathway and further clarify how metabolic reprogramming supports goblet cell-mediated barrier restoration.

Although both wild-type *A. muciniphila* and the purified Amuc_0904 protein significantly ameliorated colitis in our models, we further developed an engineered EcN (BAD−904) expressing Amuc_0904 to overcome critical translational challenges associated with native bacterial and protein therapies. First, purified protein therapeutics are often limited by low oral bioavailability due to degradation in the gastrointestinal environment and require repeated administration to maintain effective concentrations. In contrast, EcN (BAD−904) colonizes the intestinal tract and serves as a localized and continuous production system for Amuc_0904, thereby enhancing delivery efficiency and prolonging therapeutic exposure. Second, although *A. muciniphila* has probiotic benefits, its strict anaerobic nature complicates large-scale cultivation, storage, and formulation. EcN is aerotolerant, genetically tractable, and suitable for industrial production, enhancing practicality and clinical applicability. Moreover, EcN itself exhibits well-established probiotic properties, including antimicrobial activity and immune modulation,^47^^,^^48^ suggesting that the engineered strain may exert synergistic benefits beyond the activity of Amuc_0904 alone. Finally, this live bacterial delivery system aligns with growing interest in live biotherapeutic products (LBPs) and offers a cost-effective, scalable alternative to recombinant protein production.

Although our study offers a proof-of-concept for engineered EcN delivering Amuc_0904, clinical translation will require addressing key challenges. The primary hurdle is ensuring consistent production and delivery of functional protein within the human gastrointestinal tract. While EcN provides inherent advantages like gastric acid resistance and localized delivery, further optimizations-such as improving plasmid stability, fine-tuning expression with regulated promoters, or using encapsulation to enhance bacterial survival-may be necessary. Future studies focused on pharmacokinetics, long-term safety, and scalable manufacturing of this engineered bacterial therapeutic are essential next steps before clinical application.

The loss of goblet cells and thinning of the mucus layer are hallmark features of inflammatory bowel disease that contribute significantly to barrier dysfunction and disease progression. Therefore, an agent capable of directly promoting goblet cell differentiation and mucus production-as demonstrated with Amuc_0904-addresses a critical unmet therapeutic need. This approach is innovative compared to general anti-inflammatory strategies, as it targets the underlying epithelial defect responsible for disease perpetuation.

Our strategy complements other barrier-enhancing approaches currently under investigation. For instance, IL−22 therapy aims to promote epithelial regeneration and mucus production but can also provoke pro-inflammatory responses in some contexts.^49^^,^^50^ Similarly, while dietary fibers and prebiotics enhance mucus production indirectly through microbial fermentation, their efficacy depends heavily on the individual's gut microbiota composition.^51-53^ In contrast, Amuc_0904 offers a more direct and specific mechanism for restoring goblet cell function, independent of microbial metabolism. Its oral bioavailability and targeted action make it a unique therapeutic candidate worthy of further development. Furthermore, its well-defined mechanism offers a novel conceptual foundation for developing combination therapies for IBD.

Collectively, our study demonstrates that *A. muciniphila* plays a crucial role in remodeling homeostasis during colitis. Mechanistically, *A. muciniphila* OM protein Amuc_0904 directly interacts with MET on intestinal epithelia to decrease MET phosphorylation, thus inhibiting Wnt signaling and enhancing OXPHOS pathways to induce goblet cell differentiation and improve mitochondrial function. These findings shed light on a new way that *A. muciniphila* regulates intestinal homeostasis and provide a bioactive molecule for potential treatment of IBD.

## Supplementary Material

Supplementary Material Supplemental information20250922yl.docx

## Data Availability

16S rRNA gene sequencing data have been deposited into the NCBI Sequence Read Archive (SRA) database with the accession numbers PRJNA1106076 and PRJNA1105550. RNA-sequencing data have been deposited in NCBI’s Gene Expression Omnibus (GEO) with the accession number GSE288821. The genome sequence of AKK-TMU has been submitted to the GenBank database under the accession number CP156688.
