# Supplementary material for: Akkermansia muciniphila outer membrane protein Amuc_0904 modulates intestinal homeostasis by promoting goblet cell differentiation
Source: Gut Microbes. 2025 Dec 22;18(1):2587405. doi: 10.1080/19490976.2025.2587405 (PMC12810044; doi:10.1080/19490976.2025.2587405)
Supplement: Supplementary Material — Supplemental information20250922yl.docx [file KGMI_A_2587405_SM0380.docx]

**Supporting Information**

*Akkermansia muciniphila* outer membrane protein Amuc_0904 modulates intestinal homeostasis by promoting goblet cell differentiation

Figure S1

Figure S2

Figure S3

Figure S4

Figure S5

Figure S6

Figure S7

Figure S8

Figure S9

Figure S10

Figure S11

Figure S12

Figure S13

Table S1

Table S2

Table S3


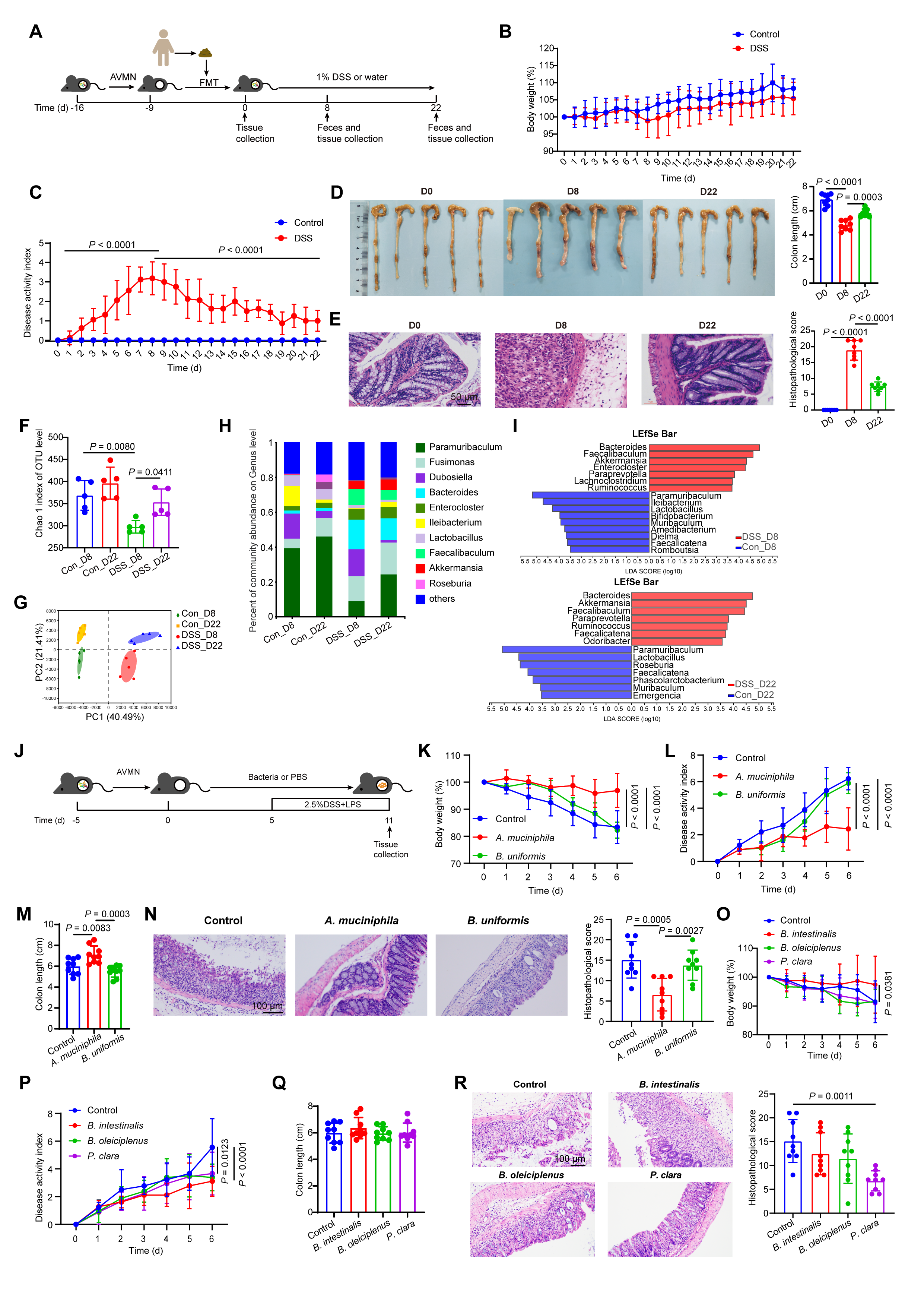


**Figure S1. *A. muciniphila* isolated from colitis recovery alleviates DSS-induced colitis.** **(A-E)** The C57BL/6J mice were subjected to AVMN treatment for 7 days, followed by fecal microbiota transplantation (FMT) from healthy volunteers for 9 days, and then exposed to 1% DSS for 22 days. **(A)** Schematic showing the experimental procedure of the FMT in addition to the DSS treatment. The body weight **(B)** and disease activity index **(C)** of the mice during DSS treatments after FMT. Analysis of the colon length **(D)**, and histological scores **(E)** of the indicated mice. **(F-I)** Bacterial DNA collected from mice feces on the 8th day and the 22nd day of DSS treatment was used to perform 16S rRNA gene sequencing. **(F)** Chao 1 index of the gut microbiota. **(G)** Principal component analysis (PCA) of the fecal microbiota. **(H)** Percentage of bacterial community abundance at the genus level in different groups. **(I)** LEfSe analysis of the different groups. **(J-R)** AVMN-treated male C57BL/6J mice were administered intragastrically with 5×10^8^ CFU of indicated bacteria for 11 days. Then, the mice were given 2.5% DSS and LPS (8 mg/kg body weight) from the 5th day of bacterial administration for another 6 days. PBS was used as the control. **(J)** Schematic for the mouse model to detect bacterial effects on DSS-induced colitis. **(K-N)** Body weight **(K)**, disease activity index **(L),** colon length **(M),** and representative H&E staining (left) and histological scores (right) of colon tissues of the indicated mice. **(O-R)** Analysis of body weight **(O)**, disease activity index **(P)**, colon length **(Q)**, and histological scores **(R)** of mice. Scale bar: 50 μm or 100 μm. *n* = 8 (B-E), *n* = 5 (F), *n* = 9 (K-R). Data are the mean ± SD. Two-way ANOVA (K-L, O-P) or one-way ANOVA (B, D-F, M-N, Q-R).


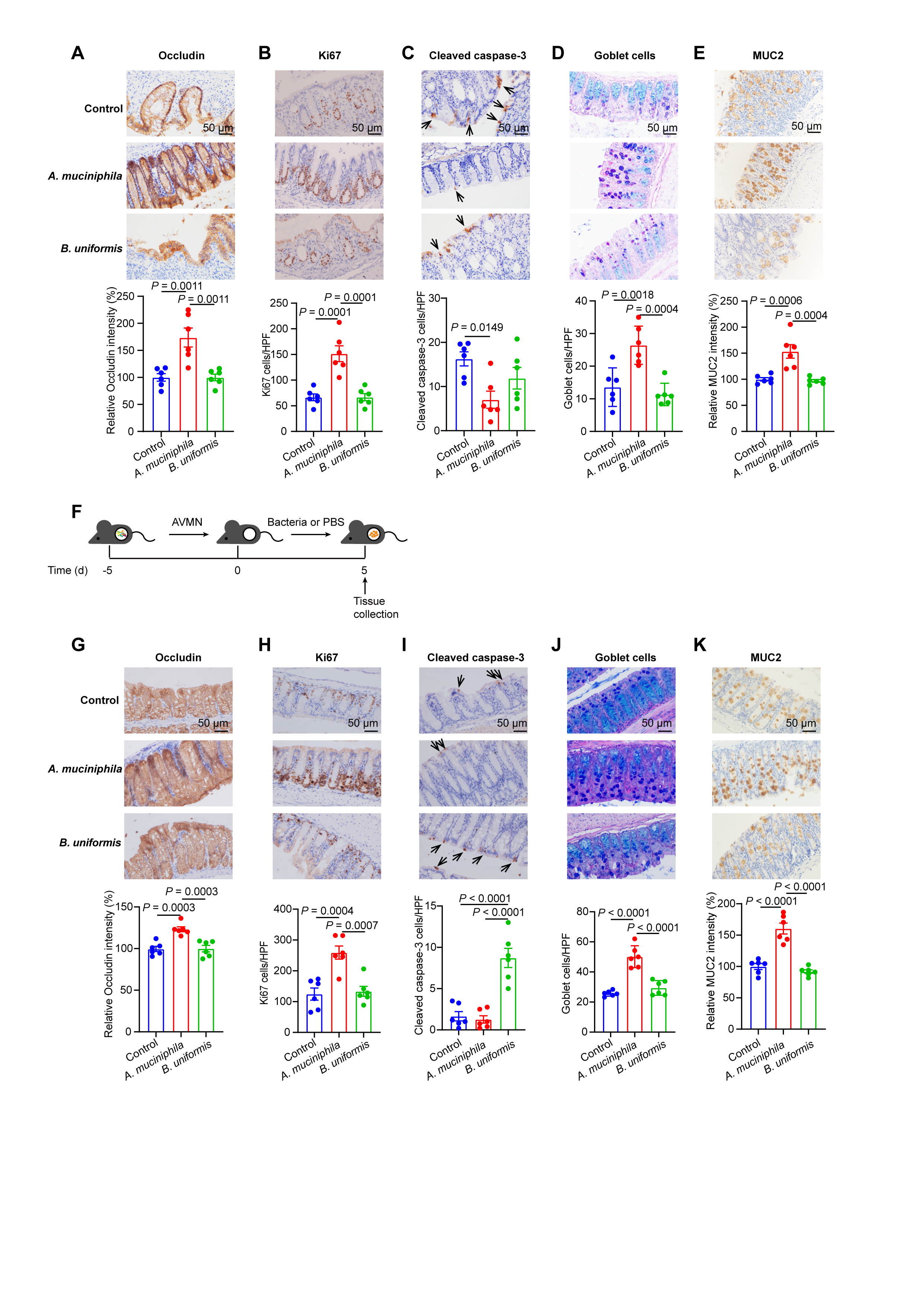


**Figure S2. *A. muciniphila* promotes goblet cell expansion and mucus formation. (A-E)** AVMN-treated male C57BL/6J mice were administered intragastrically with 5×10^8^ CFU of indicated bacteria for 11 days. Then, the mice were given 2.5% DSS and LPS (8 mg/kg body weight) from the 5th day of bacterial administration for another 6 days. Immunohistochemical staining analysis of Occludin **(A)**, Ki67 **(B)**, cleaved caspase-3 **(C),** AB/PAS staining **(D)**, and MUC2 **(E)** in colonic sections. **(F-K)** The mice were treated with 5×10^8^ CFU of *A. muciniphila* or *B. uniformis* without DSS treatment. PBS was used as the control. **(F)** Schematic diagram for the single bacterium colonization model. **(G-K)** Immunohistochemical staining analysis of Occludin **(G)**, Ki67 **(H)**, cleaved caspase-3 **(I)**, AB/PAS staining **(J)**, and MUC2 **(K)** in colon tissues. Scale bar: 50 μm. Data are the mean ± SD. *n* = 6 (A-E, G-K). One-way ANOVA (A-E, G-K).


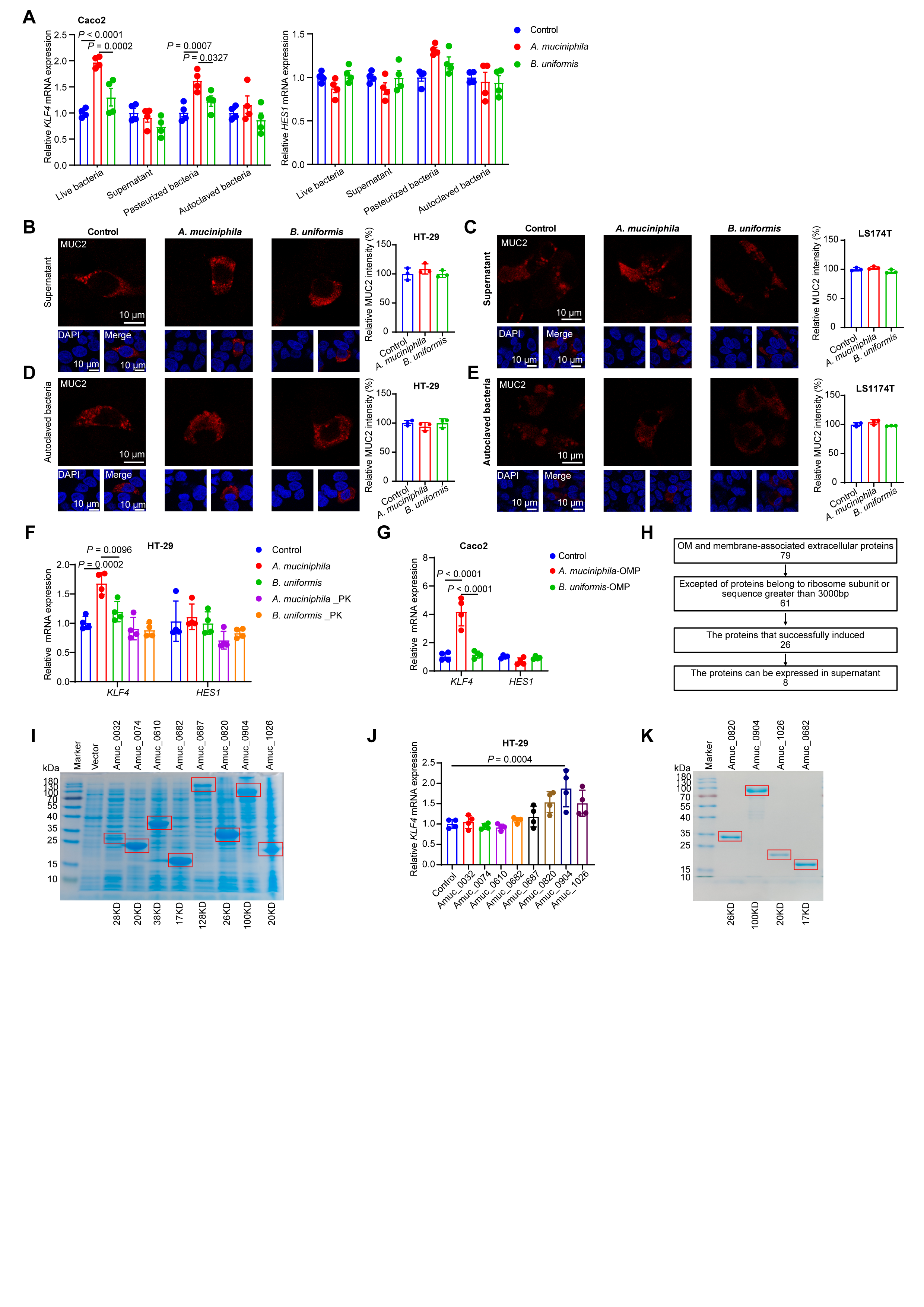


**Figure S3. Amuc_0904 promotes goblet cell characteristics and mucus production. (A)** Caco2 cells were treated with live, pasteurized, autoclaved *A. muciniphila*/*B.* *uniformis* (MOI =2), or bacteria culture supernatant (50% v/v) for 15 h. PBS or blank bacteria culture medium was used as the control. mRNA levels of *KLF4* (left) and *HES1* (right) in the indicated Caco2 cells. **(B-C)** Immunofluorescence analysis of MUC2 in HT-29 **(B)** or LS174T **(C)** treated with supernatant (20% v/v, 72 h or 48 h). **(D-E)** Immunofluorescence analysis of MUC2 in HT-29 **(D)** or LS174T **(E)** treated with autoclaved bacteria (MOI = 2, 72 h or 48 h). **(F)** mRNA levels of *KLF4* and *HES1* in HT-29 cells treated with live *A. muciniphila*/*B. uniformis* (MOI = 2, 72 h or 48 h) and bacteria treated with proteinase K (20 mg/mL, 3 h). **(G)** Caco2 were treated with outer membrane proteins of *A. muciniphila* (0.5 μg/mL, 10 h), mRNA levels of *KLF4* and *HES1* expression. **(H)** Screening process of the outer membrane proteins used in the experiment. **(I)** The SDS-PAGE and Coomassie Brilliant Blue staining of bacterial lysis supernatant over-expressing the vector, Amuc_0032, Amuc_0074, Amuc_0610, Amuc_0682, Amuc_0687, Amuc_0820, Amuc_0904, and Amuc_1026. **(J)** mRNA levels of *KLF4* in HT-29 cells treated with various outer membrane proteins (0.5 μg/mL, 72 h). **(K)** The SDS-PAGE and Coomassie Brilliant Blue staining of Ni - purified Amuc_0682, Amuc_0820, Amuc_0904, and Amuc_1026. Scale bar: 10 μm. *n* = 4 (A, F-G, J), *n* = 3 (B-E). One-way ANOVA.


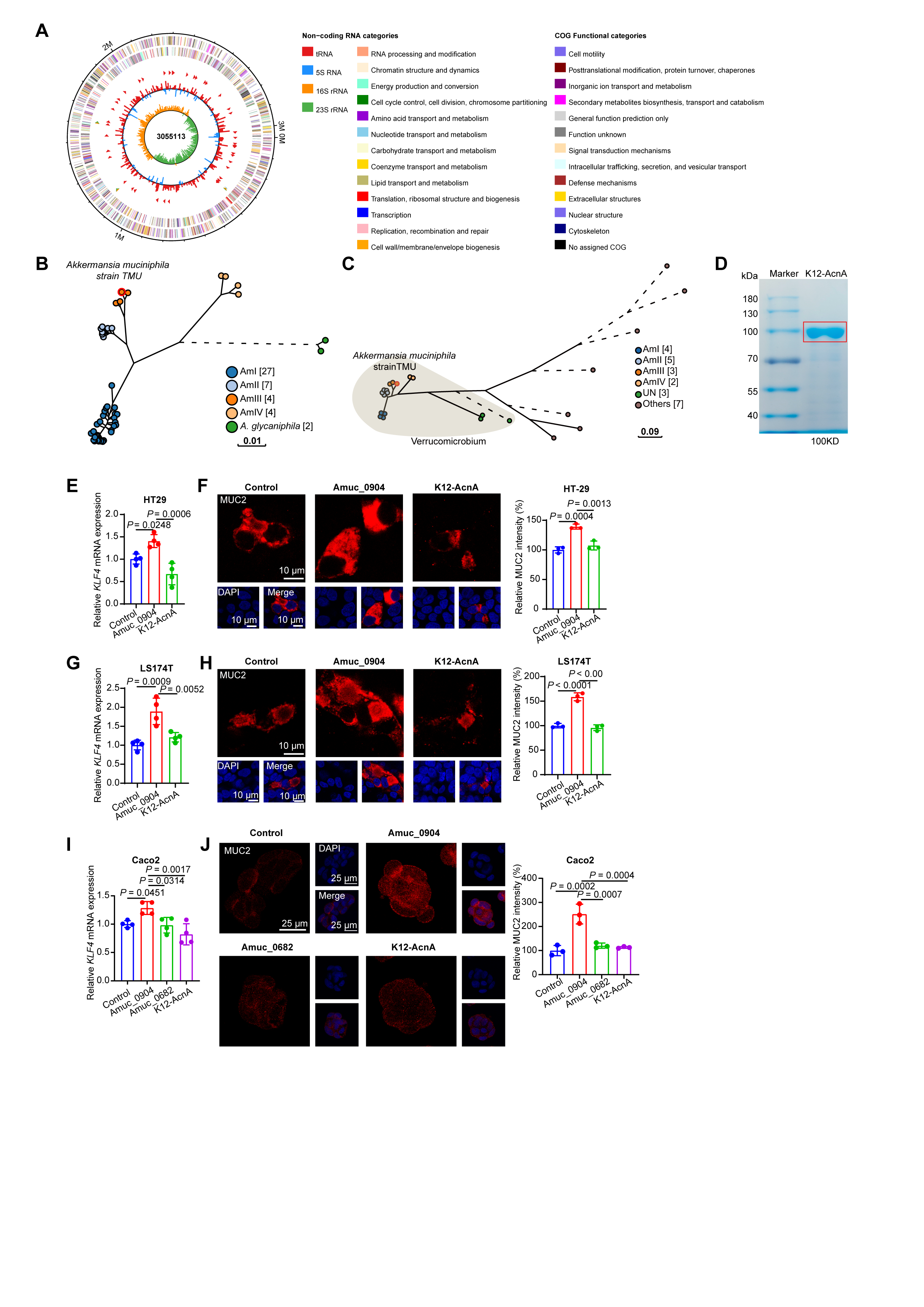


**Figure S4. Amuc_0904 specifically promotes the differentiation of goblet cells. (A)** The *A. muciniphila* TMU circular genome diagram. **(B)** The phylogenetic tree of genome sequences of *A. muciniphila* TMU and the other 43 species belong to *Akkermansia* available from GenBank. **(C)** A phylogenetic tree of Amuc_0904 homologues in representative species. **(D)** The SDS-PAGE and Coomassie Brilliant Blue staining of Ni - purified K12-AcnA. **(E-G)** HT-29 (0.5 μM, 72 h) or LS174T (0.05 μM, 48 h) were treated with Amuc_0904 or K12-AcnA, mRNA levels of *KLF4* in HT-29 cells **(E)** and LS174T cells **(G)** and immunofluorescence analysis of MUC2 in HT-29 cells **(F)** and LS174T cells **(H)**. **(I-J)** Caco2 cells were treated with Amuc_0904, Amuc_0682, or K12-AcnA (0.3 μM) for 12 h. mRNA levels of *KLF4* **(I)** and immunofluorescence analysis of MUC2 **(J)** in the indicated Caco2 cells. Scale bar: 10 μm or 25 μm. *n* = 4 (E, G, I), *n* = 3 (F, H, J). One-way ANOVA.


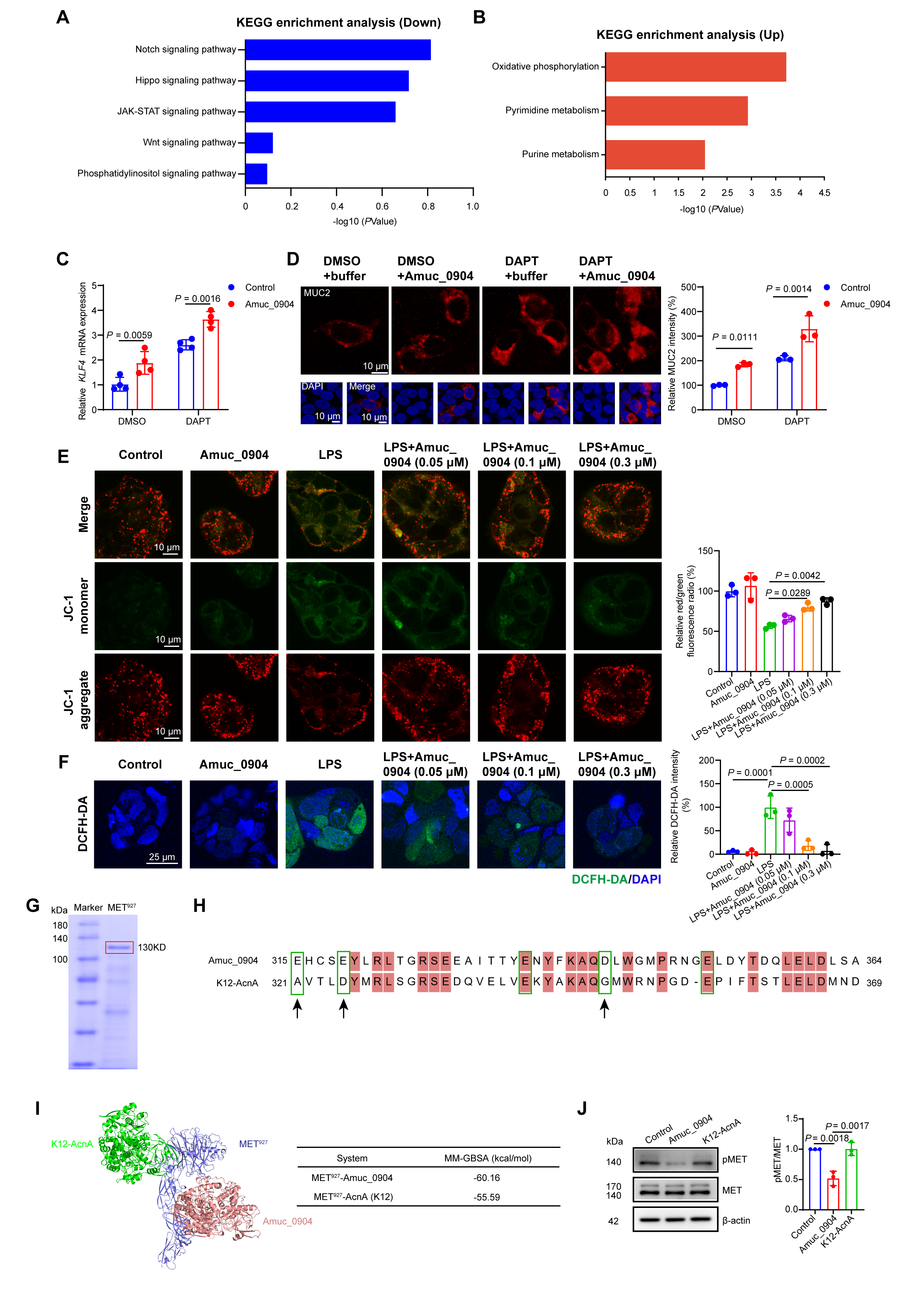


**Figure S5. Amuc_0904 regulates Wnt signaling pathway and OXPHOS pathway.** **(A-B)** KEGG enrichment analysis showing down-regulated signaling pathways **(A)** or up-regulated metabolic pathways **(B)**. **(C-D)** HT-29 cells were treated with Notch signaling pathway inhibitor DAPT (5 μM, 72 h) or DMSO in combination with or without Amuc_0904 (0.5 μM, 72 h). mRNA levels of *KLF4* **(C)** and immunofluorescence analysis of MUC2 staining **(D)** in the indicated HT-29 cells. **(E-F)** Caco2 cells were treated with LPS (1 μg/mL, 36 h) in combination with or without Amuc_0904 (0.3 μM) for 12 h. Mitochondrial membrane potential **(E)** and cellular ROS **(F)** in the indicated Caco2 cells. **(G)** The purity of GST - purified MET^927^ was assessed by SDS-PAGE and Coomassie Brilliant Blue staining. **(H)** Comparison of the amino acid sequences of Amuc_0904 from K12-AcnA. The green box represented the predicted binding sites of Amuc_0904 and MET^927^, and the arrow indicated the base differences at the binding sites. **(I)** Docking Amuc_0904 and K12-AcnA into the human MET, and the MM-GBSA (total binding free energy) of the two protein complexes (The more negative the value, the stronger the binding ability). **(J)** Western blots of pMET levels in the indicated HT-29 cells. The signal densities of pMET were normalized to those of total MET. Scale bar: 10 μm or 25 μm. Data are the mean ± SD. *n* = 3 (D-F, J), *n* = 4 (C). Two-way ANOVA (C-D) or one-way ANOVA (E-F, J).


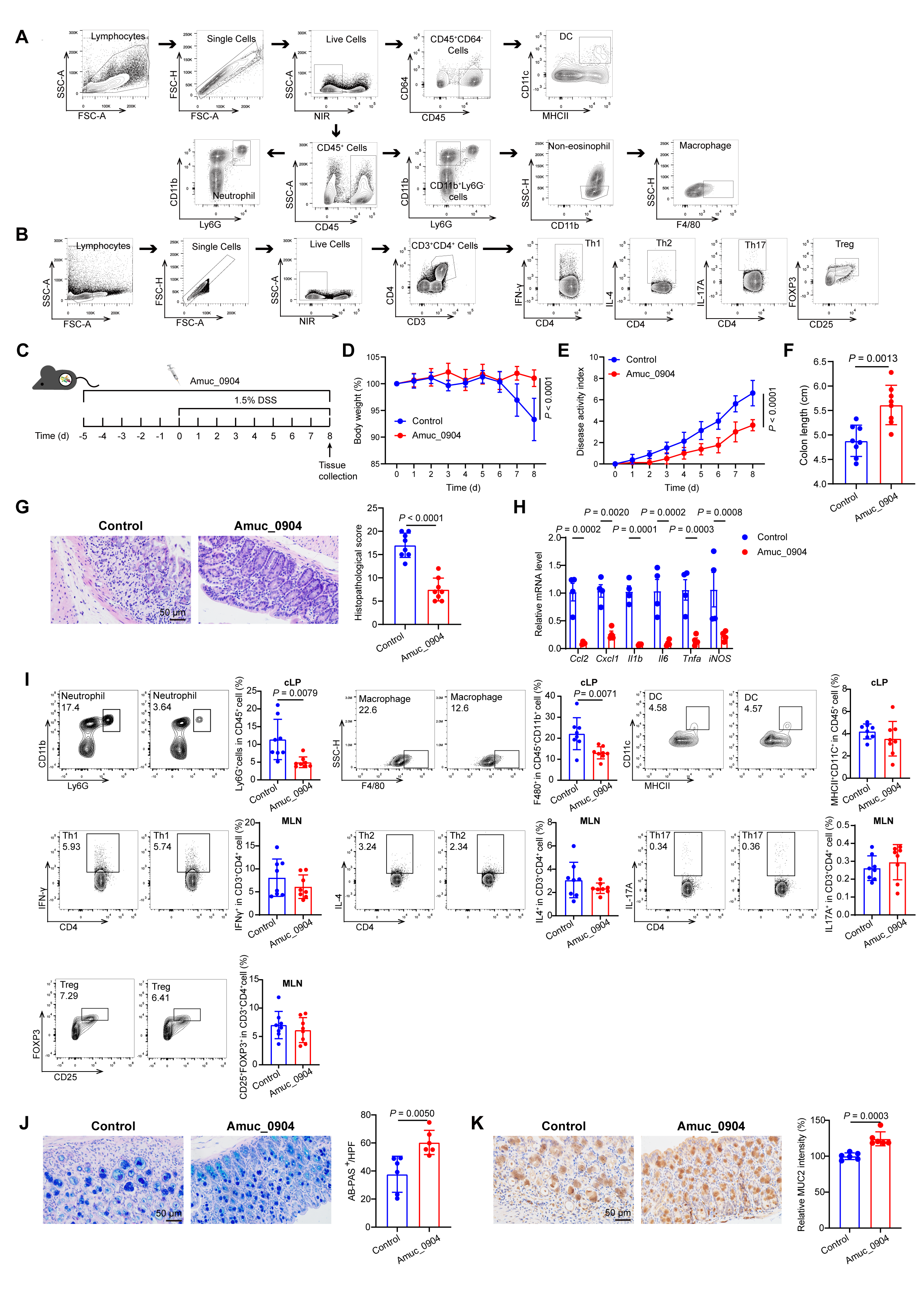


**Figure S6. Amuc_0904 has a preventive effect on DSS-induced colitis. (A-B)** Gating strategy for flow cytometry analysis of immune cells in cLP **(A)** and MLN **(B)**. **(C-K)** C57BL/6J mice were subjected to oral administration of Amuc_0904 (10 μg) for 13 days. Then, the mice were exposed to 1.5% DSS treatment from the 5th days. **(C)** Schematic diagram for the preventive model. Body weight **(D)**, DAI scores **(E)**, colon length **(F)** and histological scores **(G)** of the indicated mice. **(H)** mRNA expression levels of proinflammatory cytokines in the colon. **(I)** Representative flow cytometry analysis of the indicated cells (left) and percentage (right) of indicated cells in cLP and MLN from indicated mice. AB/PAS staining **(J)**, and immunohistochemical staining analysis of MUC2 **(K)** in colon tissues. Scale bar: 50 μm. Data are the mean ± SD. *n* = 8 (D-G, I), *n* = 4 (H), *n* = 6 (J-K). Two-way ANOVA (D-E, H) or unpaired t tests (F-G, I-K).


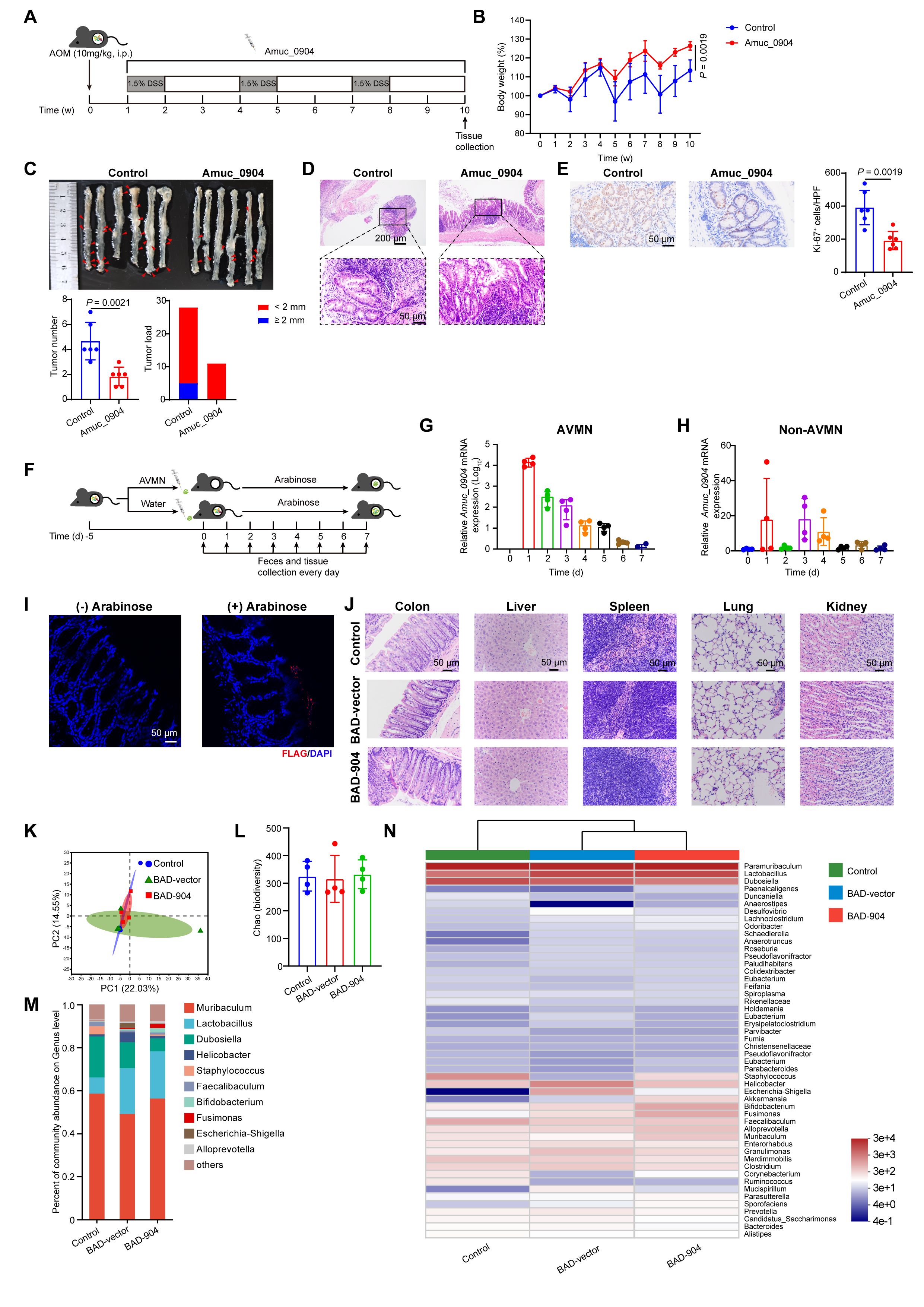


**Figure S7. Engineered EcN colonization has no effect on intestinal flora. (A-E)** WT mice were injected intraperitoneally with azoxymethane (AOM, 10 mg/kg) and treated with 1.5% DSS in drinking water for three cycles with oral administration of Amuc_0904 (10 μg per mouse). **(A)** Schematic diagram for the AOM/DSS model. **(B)** The body weight loss. **(C)** Representative photographs and quantitation of tumor number and load in colonic tissues. **(D)** Representative H&E staining of the colon tissues. **(E)** Immunohistochemical analysis of Ki-67 in colonic sections. **(F-I)** WT mice with or without AVMN treatment for 5 days were administered with 5×10^8^ CFU of EcN (BAD-vector) or EcN (BAD-904) on the first day with 2% arabinose in drinking water for one week. The feces were collected to perform realtime PCR analysis and colon tissues were collected to perform immunofluorescence analysis on the seventh day. **(F)** Schematic diagram for the colonization and induction of EcN (BAD-904). **(G-H)** mRNA levels of *Amuc_0904* in mice feces with **(G)** or without AVMN treatment **(H)** at different time points of colonization. **(I)** Immunofluorescence analysis of FLAG-Amuc_0904 in colon tissue. **(J-N)** WT mice were gavaged with 5×10^8^ CFU of EcN (BAD-904) or EcN (BAD-vector) every two days with 2% arabinose in drinking water for 5 days. The feces were collected to perform 16S rRNA gene sequencing and colon tissues were collected to perform H&E staining on the 5th day. **(J)** Representative H&E staining of the colon, liver, spleen, lung, and kidney tissues. **(K)** Principal component analysis (PCA) of the fecal microbiota. **(L)** Chao 1 index of the gut microbiota. **(M)** Percent of community abundance of bacteria at the genus level in different groups. **(N)** Heatmap showing TOP50 bacteria at the genus level. Scale bar: 200 μm or 50 μm. Data are the mean ± SD. *n =*6 (B-E), *n =*4 (G-H, K-N). Two-way ANOVA (B), one-way ANOVA (L) or unpaired t tests (C, E).


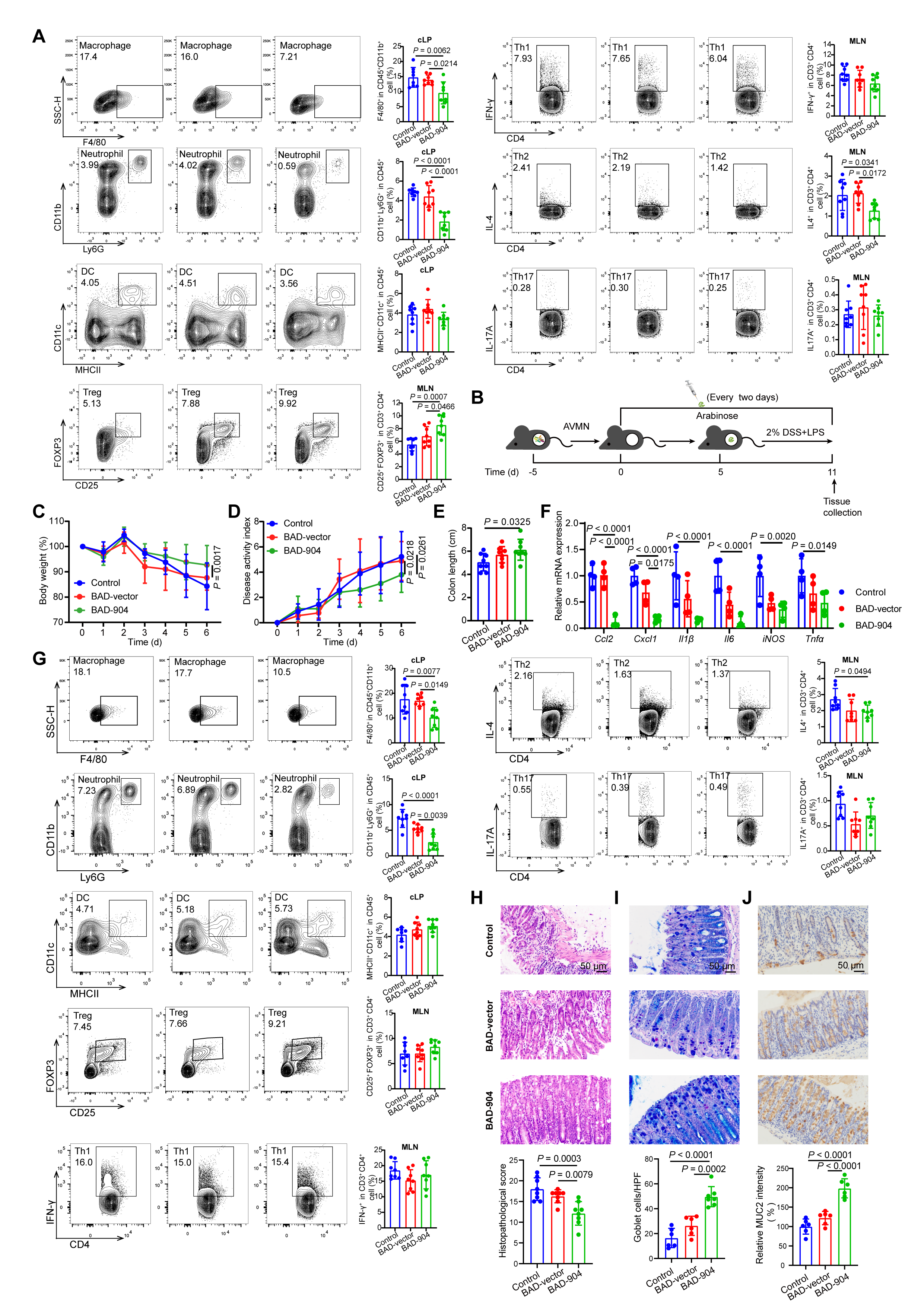


**Figure S8. EcN (BAD-904) prevents the DSS-induced colitis. (A)** Representative flow cytometry analysis of the indicated cells (left) and percentage (right) of indicated cells in MLN and cLP from indicated mice in the therapeutic model. **(B-J)** C57BL/6J mice were subjected to AVMN treatment for 5 days, followed by oral administration of EcN (BAD-904) every two days for 11 days with 2% arabinose in drinking water. Then, the mice were exposed to 2% DSS and LPS (8 mg/kg body weight) treatment from the 5th days of EcN (BAD-904) administration for 6 days. **(B)** Schematic diagram for the preventive model. Body weight **(C)**, DAI scores **(D)**, and colon length **(E)** of the indicated mice. **(F)** mRNA expression levels of proinflammatory cytokines in the colon. **(G)** Representative flow cytometry analysis from indicated mice in the preventive model. **(H-J)** H&E staining and histological **(H)**, AB/PAS staining **(I)**, and immunohistochemical staining analysis of MUC2 **(J)** in colon tissues from the indicated mice of the preventive model. Scale bar: 50 μm. Data are the mean ± SD. *n* = 8 (A, C-E, G-H), *n* = 4 (F), *n* = 6 (I-J). One-way ANOVA (A, E, G-J) or two-way ANOVA (C-D, F).


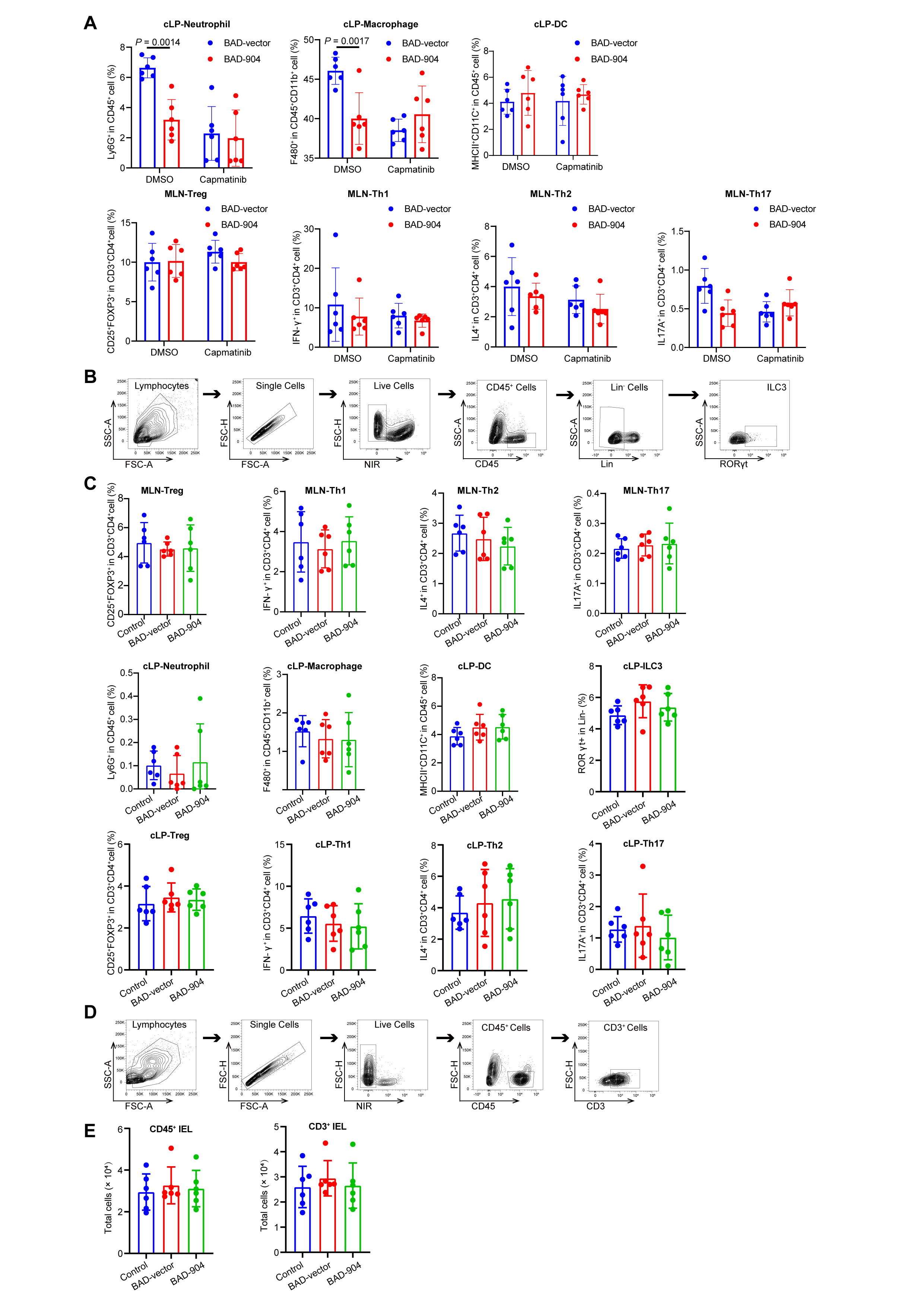


**Figure S9. EcN (BAD-904) has no effect on the proportion of immune cells under normal conditions. (A)** Percentage (right) of indicated immune cell population in cLP and MLN from indicated mice. **(B-E)** Mice were administered with engineered bacteria with 2% arabinose in drinking water for 5 days. The immune cells were collected for flow cytometry analysis. **(B)** Gating strategy for flow cytometry analysis of ILC3 in cLP. **(C)** Percentage of indicated immune cell population in MLN and cLP. **(D)** Gating strategy for flow cytometry analysis of immune cells in IEL. **(E)** Percentage of indicated immune cell population in IEL. Data are the mean ± SD. *n* = 6. Two-way ANOVA (A) or one-way ANOVA (C, E).


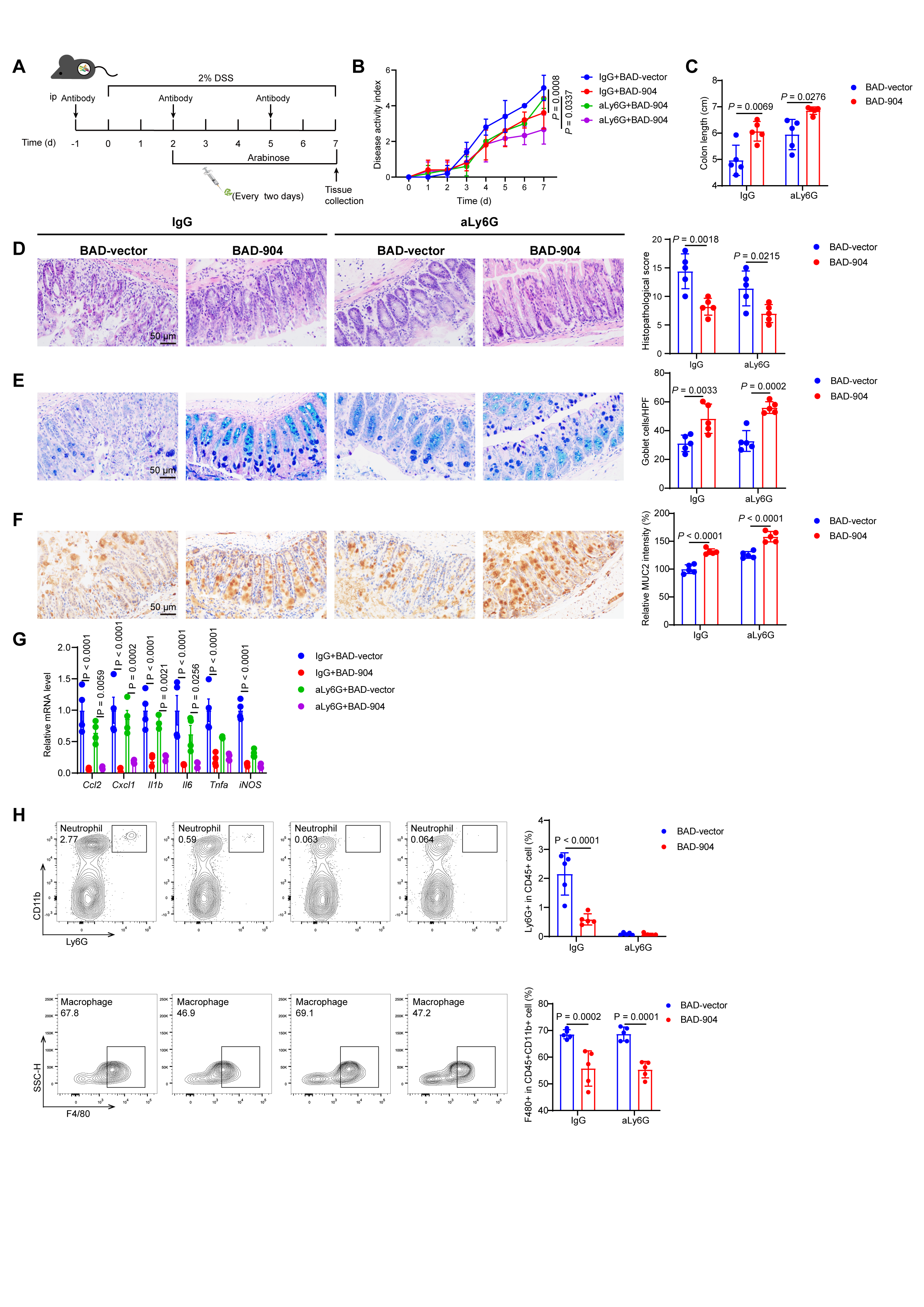


**Figure S10. The role of EcN (BAD-904) is independent of neutrophils. (A)** Schematic diagram for the neutrophil depletion experiment. After 2 days of 2% DSS treatment, the C57BL/6J mice were oral administrated EcN (BAD-904) every two days for 5 consecutive days with 2% arabinose in drinking water. 0.2 mg of anti - mouse Ly6G antibody or isotype control was intraperitoneally injected at one day before DSS administration, day 2 and day 5. **(B-C)** DAI scores **(B)**, and colon length **(C)** of the indicated mice. **(D-F)** H&E staining and histological **(D)**, AB/PAS staining **(E)**, and immunohistochemical staining analysis of MUC2 **(F)** in colon tissues. **(G)** mRNA expression levels of proinflammatory cytokines in the colon. **(H)** Percentage of neutrophils and macrophages population in cLP. Scale bar: 50 μm. Data are the mean ± SD. *n* = 5 (A-F, H), *n* = 4 (G). Two-way ANOVA.


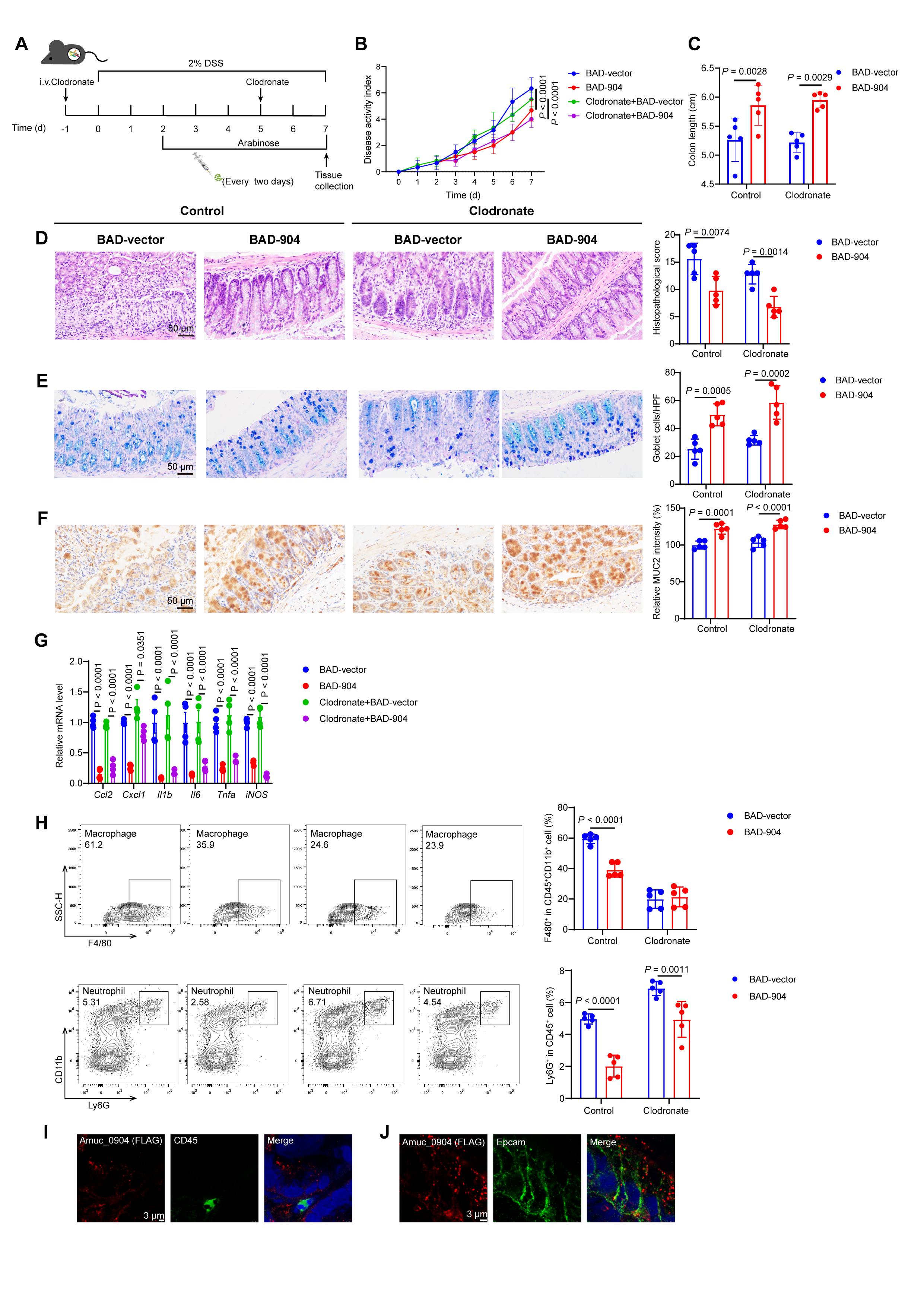


**Figure S11. The role of EcN (BAD-904) is independent of macrophages. (A)** Schematic diagram for the macrophage depletion experiment. After 2 days of 2% DSS treatment, the C57BL/6J mice were oral administrated EcN (BAD-904) every two days for 5 consecutive days with 2% arabinose in drinking water. Chlorophosphate liposome or control liposome was injected intravenously (200 μL per mouse) at one day before DSS administration and day 5. **(B-C)** DAI scores **(B)**, and colon length **(C)** of the indicated mice. **(D-F)** H&E staining and histological **(D)**, AB/PAS staining **(E)**, and immunohistochemical staining analysis of MUC2 **(F)** in colon tissues. **(G)** mRNA expression levels of proinflammatory cytokines in the colon. **(H)** Percentage of neutrophils and macrophages population in cLP. **(I-J)** Immunofluorescence staining to evaluate co-localization of Amuc_0904 (FLAG, red) and CD45 (green) **(I)** or Epcam (green) **(J)** in the colon of mice treated with EcN (BAD-904). Scale bar: 50 μm or 3 μm. Data are the mean ± SD. *n* = 5 (A-F, H), *n* = 4 (G). Two-way ANOVA.


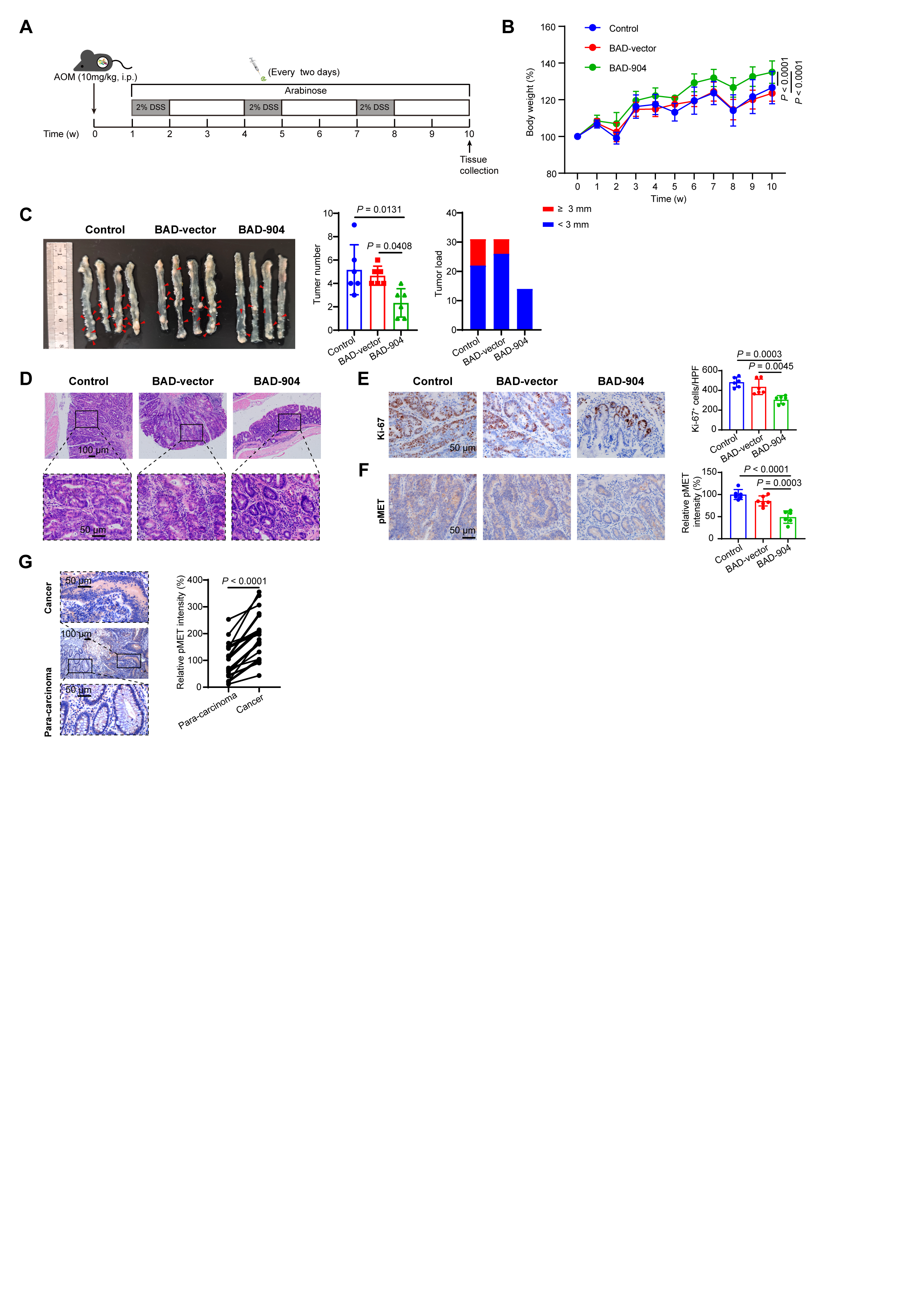


**Figure S12. EcN (BAD-904) alleviates colitis-associated colorectal cancer in mice.** WT mice were injected intraperitoneally with azoxymethane (AOM, 10 mg/kg) and treated with 2% DSS in drinking water for three cycles with oral administration of EcN (BAD-904) every two days. **(A)** Schematic diagram for the AOM/DSS model. **(B)** The body weight loss. **(C)** Representative photographs and quantitation of tumor number and load in colonic tissues. **(D)** Representative H&E staining of the colon tissues. **(E-F)** Immunohistochemical analysis of Ki-67 **(E)** and pMET **(F)** in colonic sections. **(G)** Immunohistochemical analysis of pMET in para-carcinoma and cancer tissues. Scale bar: 100 μm or 50 μm. Data are the mean ± SD. *n =*6. Two-way ANOVA (B), one-way ANOVA (C, E-F) or paired t tests (G).


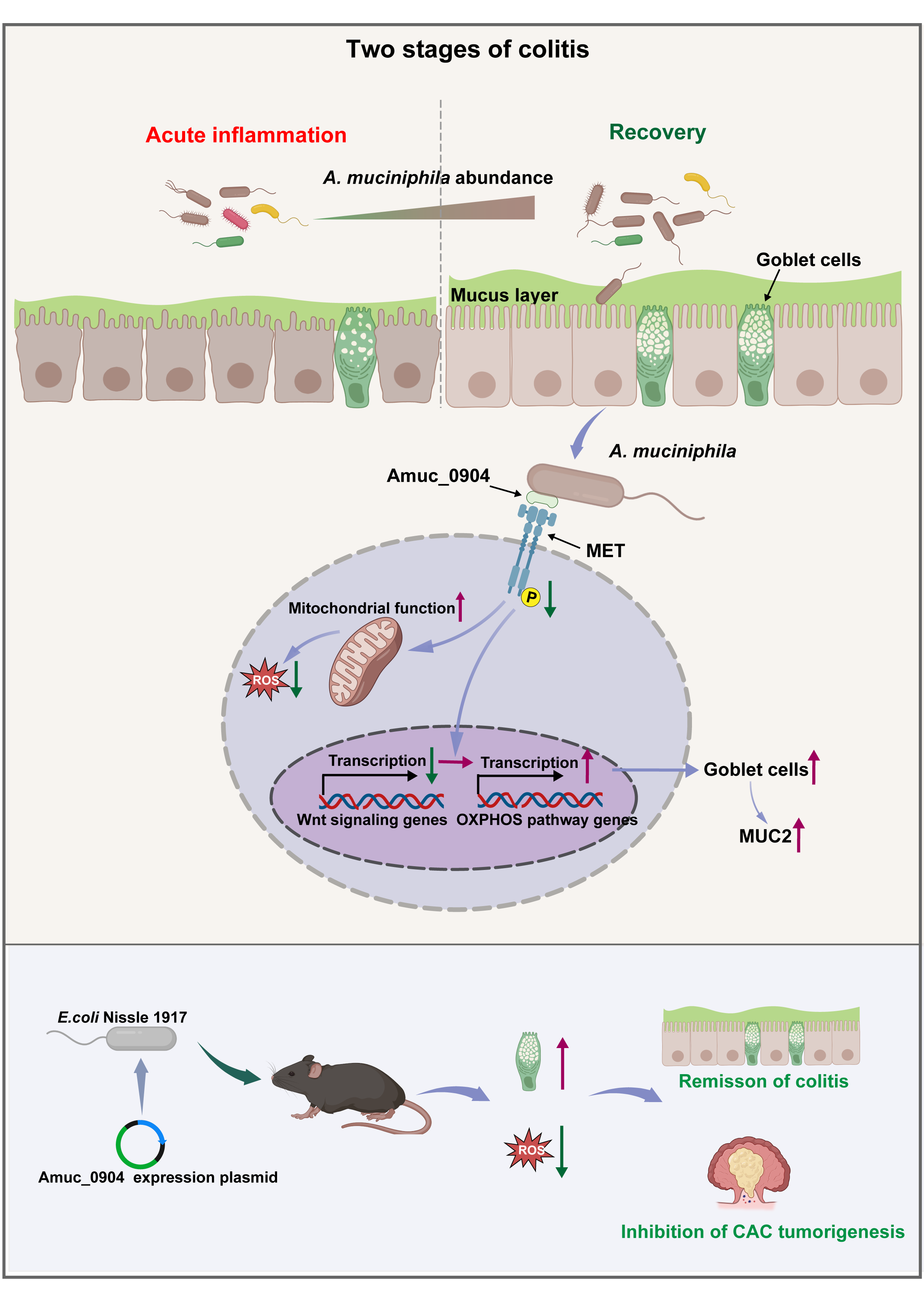


**Figure S13.** Graphical abstract illustrating the role of *A. muciniphila* in intestinal homeostasis during colitis remission. Schematic diagrams were generated by BioRender.com.

**Table S1. Characteristics of patients with UC or CRC**

| **Participants** | **Age(year)** | **Sex** | **Note** |
| --- | --- | --- | --- |
| 1 | 65 | Female | Severe UC |
| 2 | 63 | Female | Severe UC |
| 3 | 56 | Female | Severe UC |
| 4 | 32 | Female | Severe UC |
| 5 | 62 | Male | Severe UC |
| 6 | 52 | Female | Mild UC |
| 7 | 50 | Male | Mild UC |
| 8 | 24 | Male | Severe UC |
| 9 | 58 | Male | Colorectal cancer |
| 10 | 73 | Male | Colorectal cancer |
| 11 | 38 | Female | Colorectal cancer |
| 12 | 65 | Female | Colorectal cancer |
| 13 | 62 | Male | Colorectal cancer |
| 14 | 59 | Female | Colorectal cancer |
| 15 | 60 | Male | Colorectal cancer |
| 16 | 59 | Male | Colorectal cancer |
| 17 | 53 | Male | Colorectal cancer |
| 18 | 60 | Female | Colorectal cancer |
| 19 | 39 | Female | Colorectal cancer |
| 20 | 45 | Female | Colorectal cancer |
| 21 | 73 | Female | Colorectal cancer |
| 22 | 66 | Male | Colorectal cancer |
| 23 | 48 | Female | Colorectal cancer |
| 24 | 52 | Female | Colorectal cancer |
| 25 | 40 | Male | Colorectal cancer |
| 26 | 51 | Male | Colorectal cancer |
| 27 | 48 | Female | Colorectal cancer |
| 28 | 55 | Male | Colorectal cancer |
| 29 | 56 | Male | Colorectal cancer |
| 30 | 69 | Female | Colorectal cancer |
| 31 | 58 | Male | Colorectal cancer |
| 32 | 78 | Male | Colorectal cancer |

**Table S2. Prokaryotic overexpression plasmid construction and purification of putative outer membrane proteins of *A. muciniphila***

| Locus tag | Successful protein expression | Protein solubility |
| --- | --- | --- |
| Amuc_0006 | Yes | No |
| Amuc_0019 | No | No |
| Amuc_0032 | Yes | Yes |
| Amuc_0036 | No | Yes |
| Amuc_0074 | Yes | No |
| Amuc_0105 | Yes | No |
| Amuc_0172 | No | No |
| Amuc_0194 | Yes | No |
| Amuc_0219 | No | No |
| Amuc_0294 | No | No |
| Amuc_0301 | No | No |
| Amuc_0304 | No | No |
| Amuc_0308 | No | No |
| Amuc_0336 | Yes | No |
| Amuc_0355 | No | No |
| Amuc_0360 | No | No |
| Amuc_0371 | No | No |
| Amuc_0385 | Yes | No |
| Amuc_0392 | No | No |
| Amuc_0394 | Yes | No |
| Amuc_0433 | No | No |
| Amuc_0435 | Yes | No |
| Amuc_0438 | No | No |
| Amuc_0513 | No | No |
| Amuc_0576 | No | No |
| Amuc_0584 | No | No |
| Amuc_0609 | Yes | No |
| Amuc_0610 | Yes | Yes |
| Amuc_0682 | Yes | Yes |
| Amuc_0687 | Yes | Yes |
| Amuc_0735 | No | No |
| Amuc_0789 | No | No |
| Amuc_0815 | No | No |
| Amuc_0820 | Yes | Yes |
| Amuc_0823 | Yes | No |
| Amuc_0837 | No | No |
| Amuc_0904 | Yes | Yes |
| Amuc_0931 | No | No |
| Amuc_0967 | No | No |
| Amuc_1008 | No | No |
| Amuc_1026 | Yes | Yes |
| Amuc_1039 | No | No |
| Amuc_1053 | Yes | No |
| Amuc_1061 | Yes | No |
| Amuc_1061 | Yes | No |
| Amuc_1098 | Yes | No |
| Amuc_1114 | No | No |
| Amuc_1283 | No | No |
| Amuc_1310 | Yes | No |
| Amuc_1333 | No | No |
| Amuc_1382 | No | No |
| Amuc_1412 | Yes | No |
| Amuc_1420 | Yes | No |
| Amuc_1434 | Yes | No |
| Amuc_1439 | Yes | No |
| Amuc_1500 | Yes | No |
| Amuc_1512 | No | No |
| Amuc_1514 | No | No |
| Amuc_1525 | No | No |
| Amuc_1537 | No | No |
| Amuc_1583 | Yes | No |
| Amuc_1620 | No | No |
| Amuc_1638 | No | No |
| Amuc_1656 | No | No |
| Amuc_1684 | No | No |
| Amuc_1687 | Yes | No |
| Amuc_1722 | No | No |
| Amuc_1723 | No | No |
| Amuc_1743 | Yes | No |
| Amuc_1891 | No | No |
| Amuc_1939 | No | No |
| Amuc_2043 | Yes | No |
| Amuc_2077 | No | No |
| Amuc_2099 | Yes | No |
| Amuc_2107 | No | No |
| Amuc_2108 | No | No |
| Amuc_2111 | No | No |
| Amuc_2127 | No | No |
| Amuc_2152 | No | No |
| Amuc_2165 | Yes | No |

**Table S3. Reagents or oligonucleotides used in this study**

| **REAGENT or RESOURCE** | **SOURCE** | **IDENTIFIER** | | |
| --- | --- | --- | --- | --- |
| **Antibodies** | | | | |
| Anti-mouse CD45-APC | Biolegend | Cat# 103111; RRID: AB_312976 | | |
| Anti-mouse CD11b-PE-Cy7 | Invitrogen | Cat# 25-0112-81 RRID: AB_469587 | | |
| Anti-mouse F4/80-FITC | eBioscience | Cat# 11-4801-85 RRID: AB_2637192 | | |
| Anti-mouse CD45-APC-Cy7 | Biolegend | Cat# 103116 RRID: AB_312981 | | |
| Anti-mouse CD11c-APC | Biolegend | Cat# 117309; RRID: AB_ 313778 | | |
| Anti-mouse MHCII-PE | Biolegend | Cat# 107607 RRID: AB_313322 | | |
| Anti-mouse CD11c-APC | Biolegend | Cat# 117309; RRID: AB_313778 | | |
| anti-mouse CD45-PerCP/Cyanine5.5 | Biolegend | Cat# 103132 RRID: AB_893344 | | |
| anti-mouse Ly-6G-APC | Biolegend | Cat# 127614 RRID: AB_1877163 | | |
| Anti-mouse CD3-FITC | Biolegend | Cat# 100204; RRID: AB_312661 | | |
| Anti-mouse CD4-PE-Cy7 | Biolegend | Cat# 100422 RRID: AB_312706 | | |
| Anti-mouse IL-17A-APC | Biolegend | Cat# 506916 RRID: AB_536017 | | |
| Anti-mouse IFN-γ-PerCP/Cy5.5 | Invitrogen | Cat# 45-7311-82 RRID: AB_1107020 | | |
| Anti-mouse IL-4-PE | Biolegend | Cat# 504103 RRID: AB_315317 | | |
| Anti-mouse CD25-PE | Biolegend | Cat# 102008; RRID: AB_ 312857 | | |
| Anti-mouse Foxp3-APC  Anti-mouse CD45-PE  Anti-mouse Lin-FITC  Anti-mouse RORγt-APC | Biolegend  Biolegend  Invitrogen  Invitrogen | Cat# 320014; RRID: AB_ 439750  Cat# 103106; RRID: AB_312971  Cat# 22-7770-72; RRID: AB_2644066  Cat# 17-6981-82; RRID: AB_2573254 | | |
| β-Actin antibody | Abcam | Cat# ab8226 RRID: AB_306371 | | |
| MUC2 antibody | Proteintech | Cat# 27675-1-AP RRID: AB_2880943 | | |
| FLAG antibody | Sigma-Aldrich | Cat# F1804-50UG RRID: AB_262044 | | |
| Met (D1C2) antibody | Cell Signaling Technology | Cat# 8198 RRID: AB_10858224 | | |
| Phospho-Met (Tyr1234/1235) (D26) antibody | Cell Signaling Technology | Cat# 3077 RRID: AB_2143884 | | |
| β-catenin antibody | Cell Signaling Technology | Cat# 8480 RRID: AB_11127855 | | |
| Non-phospho (Active) β-catenin (Ser33/37/Thr41) (D13A1) antibody | Cell Signaling Technology | Cat# 8814 RRID: AB_11127203 | | |
| Anti-MYC antibody antibody | Cell Signaling Technology | Cat#2278 RRID: AB_490778 | | |
| Ki67 antibody | Abcam | Cat# ab16667 RRID: AB_302459 | | |
| Occludin antibody | Abcam | Cat# ab216327 RRID: AB_2737295 | | |
| Cleaved caspase-3 antibody | Cell Signaling Technology | Cat# 9664 RRID: AB_2070042 | | |
| Alexa Fluor 488 goat anti-rabbit IgG | Proteintech | Cat# SA00013-2 RRID: AB_2797132 | | |
| Alexa Fluor 594 goat anti-rabbit IgG | Proteintech | Cat# SA00013-4 RRID: AB_2810984 | | |
| Alexa Fluor 488 goat anti-Mouse IgG | Proteintech | Cat# SA00013-1 RRID: AB_2810983 | | |
| Alexa Fluor 594 goat anti-Mouse IgG | Proteintech | Cat# SA00013-3 RRID: AB_2797133 | | |
| HRP-labeled secondary antibody | Zsbio | Cat# PV-6001 RRID: AB_2864333 | | |
| Anti-Mouse IgG | SeraCare | Cat#5220-0341 RRID: AB_2891080 | | |
| Anti-Rabbit IgG | Seracare | Cat# 5220-0336 RRID: AB_2857917 | | |
| Epcam antibody | Proteintech | Cat# 21050-1-AP RRID:AB_2920805 | | |
| FITC anti-mouse CD45 antibody | Biolegend | Cat# 103108 RRID:AB_1283443 | | |
| anti - mouse Ly6G antibody | BioLegend | Cat# 127649 | | |
| **Bacterial Strains and plasmid** | | | | |
| *Akkermansia muciniphila TMU* | This paper | N/A | | |
| *Bacteroides uniformis* | This paper | N/A | | |
| *Bacteroides intestinalis* | This paper | N/A | | |
| *Bacteroides oleiciplenus* | This paper | N/A | | |
| *Paraprevotella clara* | This paper | N/A | | |
| *E. coli* K12 | Lab stored | N/A | | |
| *E. coli* Nissle 1917 | Biobw | Cat# bio-089890 | | |
| *E. coli* BL21(DE3) | CWBio | Cat# CW0809S | | |
| *E. coli* Rosetta（DE3） | ZOMANBIO | Cat# ZC125-1 | | |
| *E. coli* DH5α（DE3） | CWBio | Cat# CW0808S | | |
| pET-28a | Lab stored | N/A | | |
| pET-28a-FLAG-0904 | This paper | N/A | | |
| pET-28a-FLAG-0682 | This paper | N/A | | |
| pET-28a-FLAG-1026 | This paper | N/A | | |
| pET-28a-FLAG-0820 | This paper | N/A | | |
| pET-28a-FLAG-0032 | This paper | N/A | | |
| pET-28a-FLAG-0074 | This paper | N/A | | |
| pET-28a-FLAG-0610 | This paper | N/A | | |
| pET-28a-FLAG-0687 | This paper | N/A | | |
| pET-28a-FLAG-K12-AcnA | This paper | N/A | | |
| pGEX4T-3 | Lab stored | N/A | | |
| pGEX4T-3-MYC-MET^927^ | This paper | N/A | | |
| pTOPO-araBAD | Lab stored | N/A | | |
| pTOPO-araBAD-Ompx-904 | This paper | N/A | | |
| **Cell lines** | | | | |
| HT-29 | ATCC | Cat# HTB-38 | | |
| LS174T | ATCC | Cat# CL-188 | | |
| Caco2 | ATCC | Cat# HTB-37 | | |
| **Chemicals, peptides, and recombinant proteins** | | | | |
| Agar | Solarbio | Cat# A8190 | | |
| Bovine Serum Albumin | Sangon Biotech | Cat# A600332-0100 | | |
| DAPI Fluoromount-G | SouthernBiotech | Cat# 0100-20 | | |
| DAPI | Solarbio | Cat# C0060 | | |
| Dextran Sodium Sulfate | MP Biomedicals | Cat# 0216011090 | | |
| Azoxymethane | MP Biomedicals | Cat# 25843-45-2 | | |
| EDTA | Solarbio | Cat# E8030 | | |
| Glucose | Solarbio | Cat# G8150 | | |
| Glutamine | Solarbio | Cat# G8180 | | |
| Guanidine HCl | Solarbio | Cat# G8070 | | |
| Goat serum | Solarbio | Cat# SL038 | | |
| Lysozyme | Sangon Biotech | Cat# A610308-0005 | | |
| PBS | Biosharp | Cat# BL302A | | |
| D-PBS | Solarbio | Cat# D1040 | | |
| 1.5M Tris-HCL | Solarbio | Cat# T1010 | | |
| Tween-20 | Solarbio | Cat# T8220 | | |
| Triton X-100 | Solarbio | Cat# T8200 | | |
| Yeast extract | OXOID | Cat# LP0021B | | |
| Typtone | OXOID | Cat# LP0042B | | |
| DNase I | Thermo Scientific | Cat# EN0529 | | |
| Percoll | GE Healthcare Life Sciences | Cat# 17-0891-01 | | |
| Gentle Cell Dissociation Reagent | StemCell | Cat# 100-0485 | | |
| Matrigel | Corning | Cat# 356234 | | |
| 4% Paraformaldehyde | Solarbio | Cat# P1110 | | |
| DTT Solution | Solarbio | Cat# D1070 | | |
| IPTG | Sigma-Aldrich | Cat# I6768 | | |
| Arabinose | Sigma-Aldrich | Cat# V900920 | | |
| RIPA buffer | Solarbio | Cat# R0020 | | |
| Rotenone | MedChemExpress | Cat# HY-B1756 | | |
| FCCP | MedChemExpress | Cat# HY-100410 | | |
| Oligomycin A | MedChemExpress | Cat# HY-16589 | | |
| DAPT | MedChemExpress | Cat# HY-13027 | | |
| Lipofectamine 3000 | Thermofisher Scientific | Cat# L3000015 | | |
| OCT | Sakura Finetek | Cat# 4583 | | |
| DMSO | Solarbio | Cat# D8370 | | |
| CHIR-99021 | MedChemExpress | Cat# 252917-06-9 | | |
| Capmatinib | MedChemExpress | Cat# HY-13404 | | |
| Vancomycin | Sangon Biotech | Cat# A600983-0001 | | |
| Ampicillin | Sangon Biotech | Cat# A610028-0025 | | |
| Antimycin A | MKBio | Cat# MS0070 | | |
| Metronidazole | Sangon Biotech | Cat# A600633-0025 | | |
| Neomycin | Sangon Biotech | Cat# A610366-0025 | | |
| Ni-IDA Resin | GenScript | Cat# L00684 | | |
| Complete mini-EDTA-free protease inhibitor cocktail tablets | Roche | Cat# 11697498001 | | |
| PhosSTOP™ Phosphatase inhibitors | Roche | Cat# 04906837001 | | |
| Q5 High-Fidelity DNA Polymerase | New England Biolabs | Cat# M0491V | | |
| Collagenase | Sigma-Aldrich | Cat# C0130 | | |
| Cell Activation Cocktail (with Brefeldin A) | Biolegend | Cat# 423303 | | |
| T4 DNA Ligase | New England Biolabs | Cat# M0202S | | |
| Anti-FLAG M2 beads | Sigma | Cat# A2220 | | |
| Anti-c-MYC beads | Sigma | Cat# A7470 | | |
| Wheat Germ Agglutinin | Invitrogen | Cat# W11261 | | |
| Glutathione Sepharose 4B | Cytiva | Cat# 17075601 | | |
| RIPA buffer (high) | Solarbio | Cat# R0010 | | |
| **Critical commercial assays** | | | | |
| RevertAid First Strand cDNA Synthesis Kit | Thermo Scientific | Cat# K1621 | | |
| Ultra SYBR Mixture | CWBio | Cat# CW0957M | | |
| Western blotting membrane | Merck | Cat# ISEQ00010 | | |
| Immobilon Western Chemiluminescent HRP Substrate | Merck | Cat# WBKLS0500 | | |
| Gel Extraction Kit | Cwbio | Cat# CW2302M | | |
| AB-PAS Stain Kit | Solarbio | Cat# G1285 | | |
| EndoFree Mini Plasimid Kit | Tiangen | Cat# DP118 | | |
| TIANamp Bacteria DNA Kit | Tiangen | Cat# DP302-02 | | |
| Membrane and Cytosol Protein Extraction Kit | Beyotime | Cat# P0033 | | |
| ROS Assay Kit | Beyotime | Cat# S0033 | | |
| Total RNA Extraction Kit | Solarbio | Cat# R1200 | | |
| Rabbit Two-Step Kit (Rabbit Polymer Detection System) | ZSGB-Bio | Cat# PV-6001 | | |
| Stool DNA Kit | Omega Bio-tek | Cat# D4015-02 | | |
| Mitochondrial membrane potential assay kit with JC-1 | Beyotime | Cat# C2006 | | |
| DAB Chromogenic Kit | ZSGB-BIO | Cat# ZLI-9017 | | |
| Pierce™ BCA Protein Assay Kits | Thermofisher Scientific | Cat# 23227 | | |
| Duolink® *in situ* Proximity Ligation Assay | Sigma Aldrich | Cat# DUO92101 | | |
| Bacterial Outer Membrane Protein Extraction Kit | BestBio | Cat# BB-31512 | | |
| Chlorophosphate liposome | Target Technology, | Cat# CP-005-005 | | |
| **Oligonucleotides** | | | | |
| Mouse*-Il6* F:  AGACAAAGCCAGAGTCCTTCAG | Sangon Biotech | Custom made | | |
| Mouse*-Il6* R:  GAGCATTGGAAATTGGGGTAGG | Sangon Biotech | Custom made | | |
| Mouse*-Il1β* F:  GGGCTGGACTGTTTCTAATGC | Sangon Biotech | Custom made | | |
| Mouse*-Il1β* R:  CTTGTGACCCTGAGCGACC | Sangon Biotech | Custom made | | |
| Mouse*-Tnfα* F:  GATCGGTCCCCAAAGGGATG | Sangon Biotech | Custom made | | |
| Mouse*-Tnfα* R:  TTTGCTACGACGTGGGCTAC | Sangon Biotech | Custom made | | |
| Mouse*-iNOS* F:  GTTCTCAGCCCAACAATACAAGA | Sangon Biotech | Custom made | | |
| Mouse*-iNOS* R:  GTGGACGGGTCGATGTCAC | Sangon Biotech | Custom made | | |
| Mouse*-Cxcl1* F:  TGGCTGGGATTCACCTCAAG | Sangon Biotech | Custom made | | |
| Mouse*-Cxcl1* R:  CCGTTACTTGGGGACACCTT | Sangon Biotech | Custom made | | |
| Mouse*-β-Actin* F:  CACTGTCGAGTCGCGTCCA | Sangon Biotech | Custom made | | |
| Mouse*-β-Actin* R:  GACCCATTCCCACCATCACA | Sangon Biotech | Custom made | | |
| Mouse-*Ccl2* F: CACCAGCCAACTCTCACTGAA | Sangon Biotech | Custom made | | |
| Mouse-*Ccl2* R: CATTCCTTCTTGGGGTCAGC | Sangon Biotech | Custom made | | |
| Human-*KLF4* F:  GATGCTCACCCCACCTTCTTC | Sangon Biotech | Custom made | | |
| Human-*KLF4* R:  ACCTGGAAAATGCTCGGTCG | Sangon Biotech | Custom made | | |
| Human-*HES1* F:  TGTCAACACGACACCGGATA | Sangon Biotech | Custom made | | |
| Human-*HES1* R:  ACCTCGGTATTAACGCCCTC | Sangon Biotech | Custom made | | |
| Human-*GAPDH*-F:  ACGGATTTGGTCGTATTGGG | Sangon Biotech | Custom made | | |
| Human-*GAPDH*-R:  TGATTTTGGAGGGATCTCGC | Sangon Biotech | Custom made | | |
| Cloning Amuc_0904 into pET-28a F:  CGCGGATCCGATTACAAGGATGACGACGATAAGATGACCAAGATTGCCAAG | Sangon Biotech | Custom made | |  |
| Cloning Amuc_0904 into pET-28a R:  CCGCTCGAGTTTGAGGATTTGAGTGAGGAC | Sangon Biotech | Custom made | |  |
| Cloning Amuc_0820 into pET-28a F:  CCGGAATTCGATTACAAGGATGACGACGATAAGATGGGGTGGACGTCCTGC | Sangon Biotech | Custom made | |  |
| Cloning Amuc_0820 into pET-28a R:  CCGCTCGAGCTGTACGGCAGGGGCTTTCTG | Sangon Biotech | Custom made | |  |
| Cloning Amuc_0032 into pET-28a F:  CGCGGATCCGATTACAAGGATGACGACGATAAGATGAGGCTGTTTCCCATC | Sangon Biotech | Custom made | |  |
| Cloning Amuc_0032 into pET-28a R:  CCGCTCGAGAAAAACGTAAGAGCACCAAAGG | Sangon Biotech | Custom made | |  |
| Cloning Amuc_0074 into pET-28a F:  CGCGGATCCGATTACAAGGATGACGACGATAAGATGAACAAACTCCTGCTTCCC | Sangon Biotech | Custom made | |  |
| Cloning Amuc_0074 into pET-28a R:  CCGCTCGAGGCGGACCGGGAGGGAGGT | Sangon Biotech | Custom made | |  |
| Cloning Amuc_0610 into pET-28a F:  CGCGGATCCGATTACAAGGATGACGACGATAAGATGAATGTGAAAAAGCTGCTTG | Sangon Biotech | Custom made | |  |
| Cloning Amuc_0610 into pET-28a R:  CCGCTCGAGTCTGCTGGTGACGCCCAG | Sangon Biotech | Custom made | |  |
| Cloning Amuc_0682 into pET-28a F:  CGCGGATCCGATTACAAGGATGACGACGATAAGATGCTGGAAGCCTACACG | Sangon Biotech | Custom made | |  |
| Cloning Amuc_0682 into pET-28a R:  CCGCTCGAGAAAGTCCAGGTACATTTCCGC | Sangon Biotech | Custom made | |  |
| Cloning Amuc_0687 into pET-28a F:  CTAGCTAGCGATTACAAGGATGACGACGATAAGATGAAATTAAGACTCCCACATATG | Sangon Biotech | Custom made | |  |
| Cloning Amuc_0687 into pET-28a R:  CCGCTCGAGGAACGTATAGCTGATGGCTCC | Sangon Biotech | Custom made | |  |
| Cloning Amuc_1026 into pET-28a F:  CGCGGATCCGATTACAAGGATGACGACGATAAGATGGCAACTGACAAGAATATC | Sangon Biotech | Custom made | |  |
| Cloning Amuc_1026 into pET-28a R:  CCGCTCGAGTTTGTCCAGAACCTTGTTCCA | Sangon Biotech | Custom made | |  |
| Cloning K12-AcnA into pET-28a F:  CAAATGGGTCGCGGATCCGATTACAAGGACGACGATGACAAGATGTCGTCAACCCTACGAGA | Sangon Biotech | Custom made | |  |
| Cloning K12-AcnA into pET-28a R:  GTGGTGGTGGTGCTCGAGCTTCAACATATTACGAATGA | Sangon Biotech | Custom made | |  |
| qRT-PCR for Amuc_0904 F:  AGCCAGGAAGCGGAATTGAA | Sangon Biotech | Custom made | |  |
| qRT-PCR for Amuc_0904 R:  GTTTCAAAACCCAGGGCGTC | Sangon Biotech | Custom made | |  |
| Mouse*-Muc2* F:  AGAAGCCAGATCCCGAAACC | Sangon Biotech | Custom made | |  |
| Mouse*-Muc2* R:  TTGTAGGAGTCTCGGCAGTCAG | Sangon Biotech | Custom made | |  |
| qRT-PCR for bacteria 517F:  GCCAGCAGCCGCGGTAA | Sangon Biotech | Custom made | |  |
| qRT-PCR for bacteria 798R:  AGGGTATCTAATCCT | Sangon Biotech | Custom made | |  |
| Mouse*-Lgr5* F:  CAGCCTCAAAGTGCTTATGCT | Sangon Biotech | Custom made | |  |
| Mouse*-Lgr5* R:  GTGGCACGTAACTGATGTGG | Sangon Biotech | Custom made | |  |
| Mouse*-Sox9* F:  CGGAACAGACTCACATCTCTCC | Sangon Biotech | Custom made | |  |
| Mouse*-Sox9* R:  GCTTGCACGTCGGTTTTGG | Sangon Biotech | Custom made | |  |
| Mouse*-Bmi1* F:  ATCCCCACTTAATGTGTGTCCT | Sangon Biotech | Custom made | |  |
| Mouse*-Bmi1* R:  CTTGCTGGTCTCCAAGTAACG | Sangon Biotech | Custom made | |  |
| Mouse*-Fcgbp* F:  AGGCATTCAGTGTGCATCTGG | Sangon Biotech | Custom made | |  |
| Mouse*-Fcgbp* R:  GAAGGTGGTATAGTGGGGATCA | Sangon Biotech | Custom made | |  |
| Mouse*-Klf4* F:  ATCCTTTCCAACTCGCTAACCC | Sangon Biotech | Custom made | |  |
| Mouse*-Klf4* R:  CGGATCGGATAGCTGAAGCTG | Sangon Biotech | Custom made | |  |
| Mouse*-Atoh1* F:  GAGTGGGCTGAGGTAAAAGAGT | Sangon Biotech | Custom made | |  |
| Mouse*-Atoh1* R:  GGTCGGTGCTATCCAGGAG | Sangon Biotech | Custom made | |  |
| Cloning MET^927^ into pGEX4T-3 F:  GATCTGGTTCCGCGTGGATCCA  TGAAGGCCCCCGCTGTG | Sangon Biotech | Custom made | |  |
| Cloning MET^927^ into pGEX4T-3 R:  GTCACGATGCGGCCGCTCGAGC  AAGTCCTCTTCAGAAATGAGCTTT | Sangon Biotech | Custom made | |  |
| Inserting Amuc_0904 into Ompx after a serine residue at position 53 F1:  ATGAAAAAAATTGCATGTCTTTCA | Sangon Biotech | Custom made | |  |
| Inserting Amuc_0904 into Ompx after a serine residue at position 53 R1:  CTTGGCAATCTTGGTCATGCTTGCAGTACGGCTTTTCTCG | Sangon Biotech | Custom made | |  |
| Inserting Amuc_0904 into Ompx after a serine residue at position 53 F2:  AAAAGCCGTACTGCAAGCATGACCAAGATTGCCAAGAGCAC | Sangon Biotech | Custom made | |  |
| Inserting Amuc_0904 into Ompx after a serine residue  at position 53 R2:  TTGTTGTAGTCACCAGACTTATCGTCGTCATCCTTGTAATCTTT | Sangon Biotech | Custom made | |  |
| Inserting Amuc_0904 into Ompx after a serine residue at position 53 F3:  GATGACGACGATAAGTCTGGTGACTACAACAAAAACC | Sangon Biotech | Custom made | |  |
| Inserting Amuc_0904 into Ompx after a serine residue at position 53 R3:  GAAGCGGTAACCAACACC | Sangon Biotech | Custom made | |  |
| Cloning Ompx-Amuc_0904 into  pTOPO-BAD F:  ATAAAAAATGGAATTCATGAAAAAAATTGCATGTCTTTCAG | Sangon Biotech | Custom made | |  |
| Cloning Ompx-Amuc_0904 into  pTOPO-BAD R:  ATGCTTGTTATCGTAAAGAGTTTGTAGAAACGCAAAAAGGC | Sangon Biotech | Custom made | |  |
| siScr F:  UUCUCCGAACGUGUCACGUTT | Sangon Biotech | Custom made | |  |
| siScr R:  ACGUGACACGUUCGGAGAATT | Sangon Biotech | Custom made | |  |
| si*MET* F:  GGACCAGUCCUACAUUGAUTT | Sangon Biotech | Custom made | |  |
| si*MET* R:  AUCAAUGUAGGACUGGUCCTT | Sangon Biotech | Custom made | |  |
| **Deposited data** | | | |  |
| 16S rRNA gene sequencing | SRA | PRJNA1106076  PRJNA1105550 |  |  |
| RNA sequencing data | GSE | GSE288821 |  |  |
| Bacterial whole genome sequencing | GenBank | CP156688 |  |  |
| **Software and algorithms** |  |  |  |  |
| Prism 6 | GraphPad Software | https://www.graphpad.com/  RRID: SCR_002798 |  |  |
| UniProt database | N/A |  |  |  |
| ImageJ | ImageJ | https://imagej.net/ RRID:SCR_003070 |  |  |
| FlowJo10.4 | TreeStar | https://www.flowjo.com/  RRID: SCR_008520 |  |  |
| Image Pro Plus | Media  Cybernetics | http://www.mediacy.com/imageproplus/  RRID: SCR_007369 |  |  |
